# Supplementary material for: In Vitro and In Silico Anti-Picornavirus Triterpene Alkanoic Acid Ester from Saudi Collection of Rhazya stricta Decne
Source: Metabolites. 2023 Jun 13;13(6):750. doi: 10.3390/metabo13060750 (PMC10305336; doi:10.3390/metabo13060750)
Supplement: Supplementary file 1 [file metabolites-13-00750-s001.zip › metabolites-2405213-supplementary.pdf]

metabolites

MDPI

Type of the Paper (Article, Review, Communication, etc.)

In vitro and *In silico* Anti-Picornavirus Triterpene Alkanoic Acid Ester from Saudi Collection of *Rhazya stricta* Decne.

Maged S. Abdel-Kader [1,2*], Fahad S. Almutib [3], Abdullah F. Aldosari [3], Gamal A. Soliman [4,5], Hisham Y. Elzorba [5], Mohammed H. Alqarni [1], Reham S. Ibrahim[2] and Hala H. Zaatout [2]

[1] Department of Pharmacognosy, College of Pharmacy, Prince Sattam bin Abdulaziz University, Al kharj11942, Saudi Arabia; m.youssef@psau.edu.sa , m.alqarni@psau.edu.sa

[2] Department of Pharmacognosy, College of Pharmacy, Alexandria University, Alexandria 21521, Egypt, hala.zatout@alexu.edu.eg , reham.abdelkader@alexu.edu.eg

[3] College of Pharmacy, Prince Sattam bin Abdulaziz University, Al kharj11942, Saudi Arabia; Fahadsmoteb@gmail.com , ph.abdullah77@gmail.com

[4] Department of Pharmacology, College of Pharmacy, Prince Sattam bin Abdulaziz University, Al kharj11942, Saudi Arabia; g.soliman@psau.edu.sa

[5] Department of Pharmacology, College of Veterinary Medicine, Cairo University, Giza, Egypt

* Correspondence: m.youssef@psau.edu.sa ; Tel.: +966-545-539-145

Table of Contents

Page

Figure S1. ^{1}HNMR spectrum of **1** ………………………………………………………… 4
Figure S2. ^{1}HNMR spectrum of **1** (Exp.)…………………………………………………… 4
Figure S3. ^{13}C NMR spectrum of **1** (Exp.)…………………………………………………… 5
Figure S4. ^{13}CNMR spectrum of **1** (Exp.)…………………………………………………… 5
Figure S5. DEPT135 spectrum of **1** (Exp.)…………………………………………………… 6
Figure S6. DEPT135 spectrum of **1** (Exp.) ………………………………………………… 6
Figure S7. HRESIMS spectrum of **1** ………………………………………………… 7
Figure S8. ^{1}HNMR spectrum of **1a** …………………………………………………...... 8
Figure S9. ^{1}HNMR spectrum of **1a** (Exp.)…………………………………………… 8
Figure S10. ^{1}HNMR spectrum of **1a** (Exp.)…………………………………………………… 9
Figure S11. ^{13}CNMR spectrum of **1a** (Exp.)…………………………………………………… 9
Figure S12. ^{13}CNMR spectrum of **1a** (Exp.)…………………………………………………… 10
Figure S13. DEPT135 spectrum of **1a** ……………………………………………………… 10
Figure S14. COSY spectrum of **1a** ……………………………………………………………… 11
Figure S15. HSQC spectrum of **1a** ……………………………………………………………... 11
Figure S16. HSQC spectrum of **1a** (Exp.)……………………………………………………… 12
Figure S17. HSQC spectrum of **1a** (Exp.)……………………………………………………… 12
Figure S18. HMBC spectrum of **1a** (Exp.)…………………………………………………… 13
Figure S19. HMBC spectrum of **1a** (Exp.)…………………………………………………… 13
Figure S20. HMBC spectrum of **1a** (Exp.)…………………………………………………… 14
Figure S21. HRESIMS spectrum of **1a** ………………………………………………………… 14
Figure S22. ^{1}HNMR spectrum of **1b** (Exp.)…………………………………………………… 15
Figure S23. ^{1}HNMR spectrum of **1b** (Exp.)…………………………………………………… 15
Figure S24. ^{1}HNMR spectrum of **1b** (Exp.)…………………………………………………… 16
Figure S25. ^{13}CNMR spectrum of **1b** ……………………………………………………….. 16
Figure S26. ^{13}CNMR spectrum of **1b** (Exp.) ………………………………………………… 17
Figure S27. DEPT135 spectrum of **1b** ………………………………………………………… 17
Figure S28. HSQC spectrum of **1b** ……………………………………………………………… 18

Figure S29. HSQC spectrum of **1b** (Exp.) ………………………………………………… 18
Figure S30. HSQC spectrum of **1b** (Exp.) ………………………………………………… 19
Figure S31. H2BC spectrum of **1b** ………………………………………………………… 19
Figure S32. H2BC spectrum of **1b** (Exp.) ………………………………………………… 20
Figure S33. HMBC spectrum of **1b** ………………………………………………………… 20
Figure S34. HRESIMS spectrum of **1b** (Negative mode)……………………………… 21
Figure S35. HRESIMS spectrum of **1b Ac** (Negative mode)………………………… 21
Figure S36. ^{1}HNMR spectrum of **2** (Exp.)………………………………………………. 22
Figure S37. ^{1}HNMR spectrum of **2** (Exp.)………………………………………………. 22
Figure S38. ^{13}CNMR spectrum of **2** (Exp.)……………………………………………… 23
Figure S39. ^{13}CNMR spectrum of **2** (Exp.)……………………………………………… 23
Figure S40. DEPT135 spectrum of **2** (Exp.) ……………………………………… 24
Figure S41. DEPT135 spectrum of **2** (Exp.) ……………………………………… 24
Figure S42. HSQC spectrum of **2** (Exp.) ………………………………………… 25
Figure S43. H2BC spectrum of **2** (Exp.) ………………………………………………. 25
Figure S44. HMBC spectrum of **2** ………………………………………………………… 26
Figure S45. HRESIMS spectrum of **2** (Positive mode) ………………………………. 26
Figure S46. ^{1}HNMR spectrum of **2a** ……………………………………………………… 27
Figure S47. ^{1}HNMR spectrum of **2a** ……………………………………………………… 27
Figure S48. ^{13}CNMR spectrum of **2a** (Exp.)………………………………………………. 28
Figure S49. ^{13}CNMR spectrum of **2a** (Exp.)………………………………………………. 28
Figure S50. DEPT135 spectrum of **2a** (Exp.)……………………………………………. 29
Figure S51. DEPT135 spectrum of **2a** (Exp.)……………………………………………. 29
Figure S52. COSY spectrum of **2a** ………………………………………………………… 30
Figure S53. HSQC spectrum of **2a** (Exp.)………………………………………………… 30
Figure S54. HSQC spectrum of **2a** (Exp.)………………………………………………… 31
Figure S55. H2BC spectrum of **2a** (Exp.)…………………………………………………. 31

Figure S56. H2BC spectrum of **2a** (Exp.)………………………………………………….. **32**
Figure S57. HMBC spectrum of **2a** (Exp.)…………………………………………………. **32**
Figure S58. HMBC spectrum of **2a** (Exp.)…………………………………………………. **33**
Figure S59. HRESIMS spectrum of **2a** (Positive mode) ………………………………… **33**
Figure S60. 3D and 2D interaction diagrams of FMDV 3C^{pro} with glycyrrhizic acid in 2D representation (lower panel). **34**
Figure S61. 3D and 2D interaction diagrams of FMDV 3Cpro with ribavirin in 2D representation (lower panel). **35**
Table S1. Determination of total extract and fractions of *R. stricta* cytotoxicity on BHK cell. **36**
Table S2. Determination of compounds **1-9** cytotoxicity on BHK cell. **38**

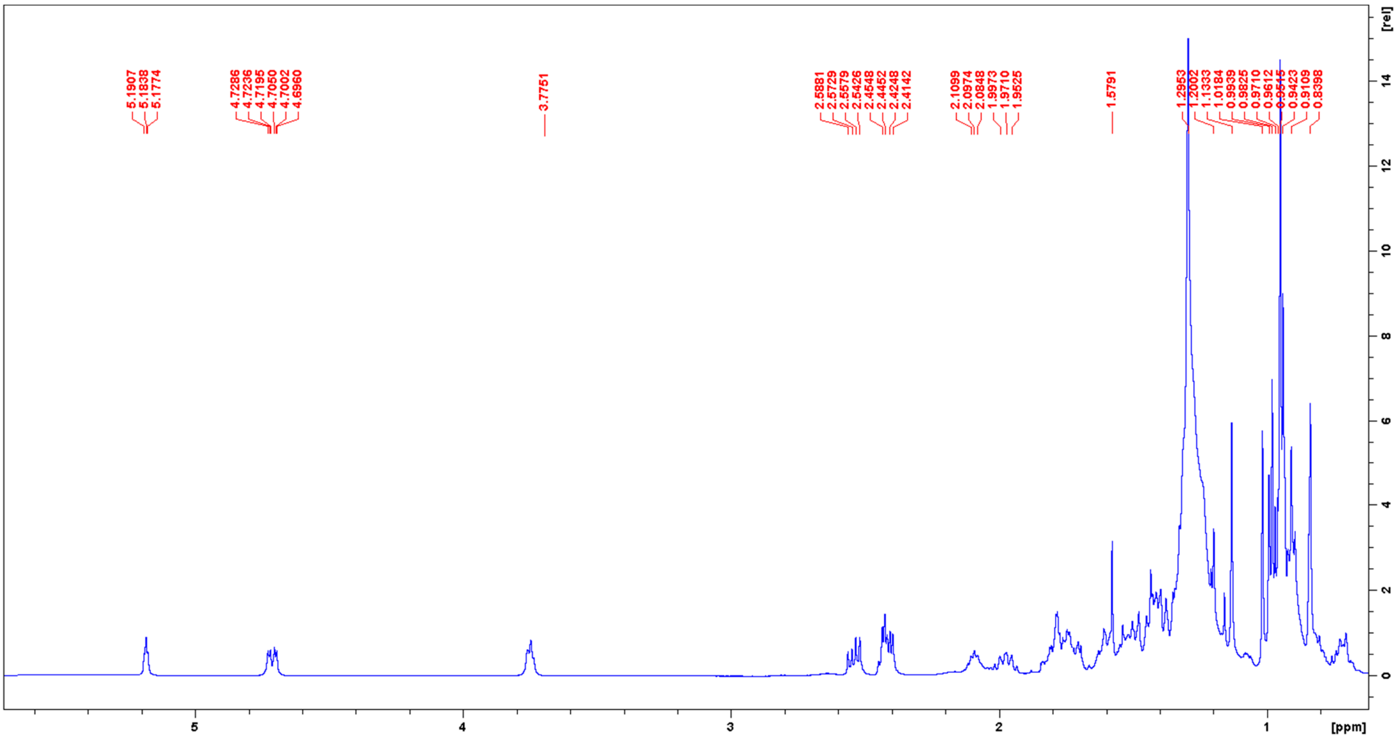

Figure S1. ^{1}HNMR spectrum of **1.**

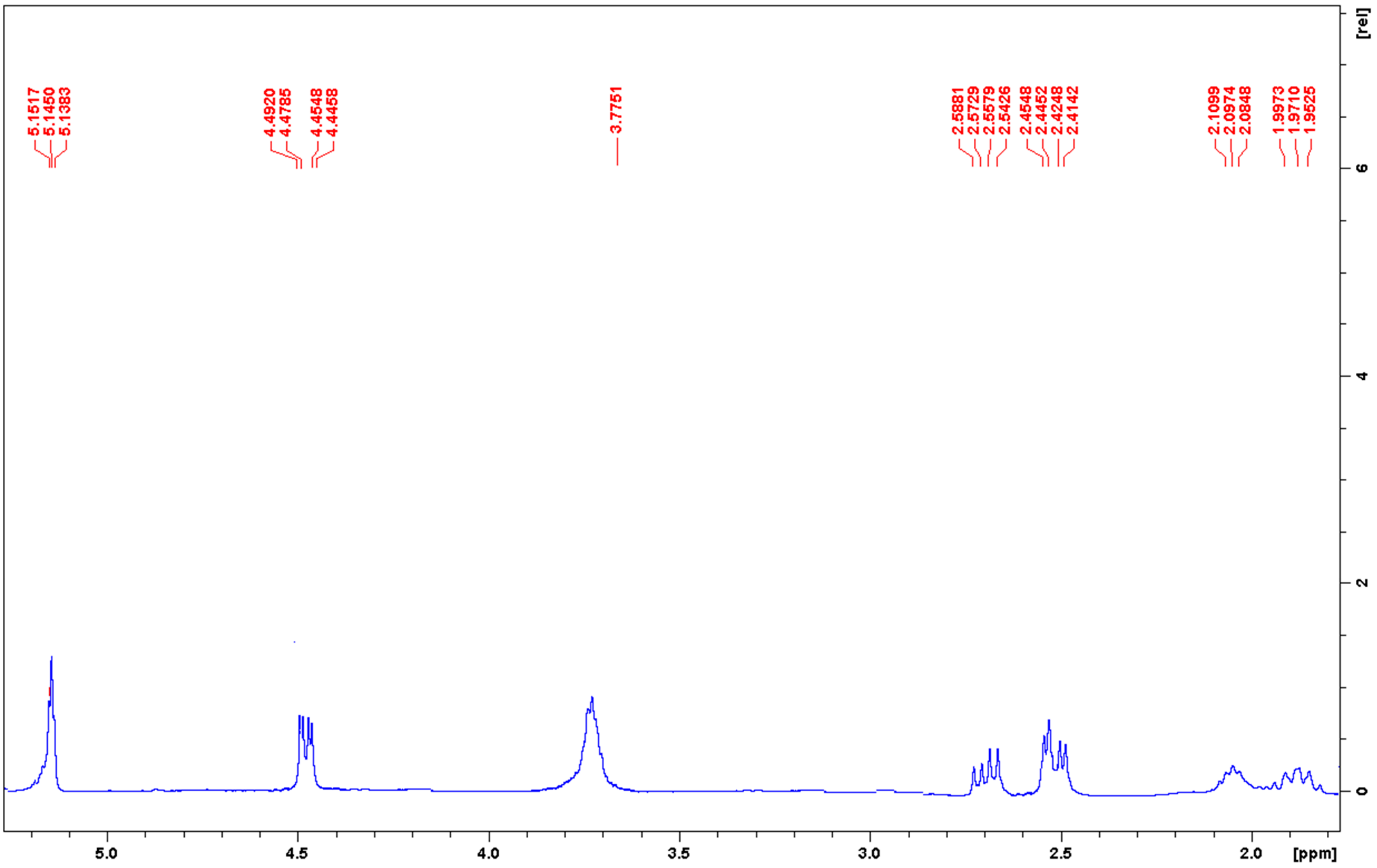

Figure S2. ^{1}HNMR spectrum of **1**(Exp.).

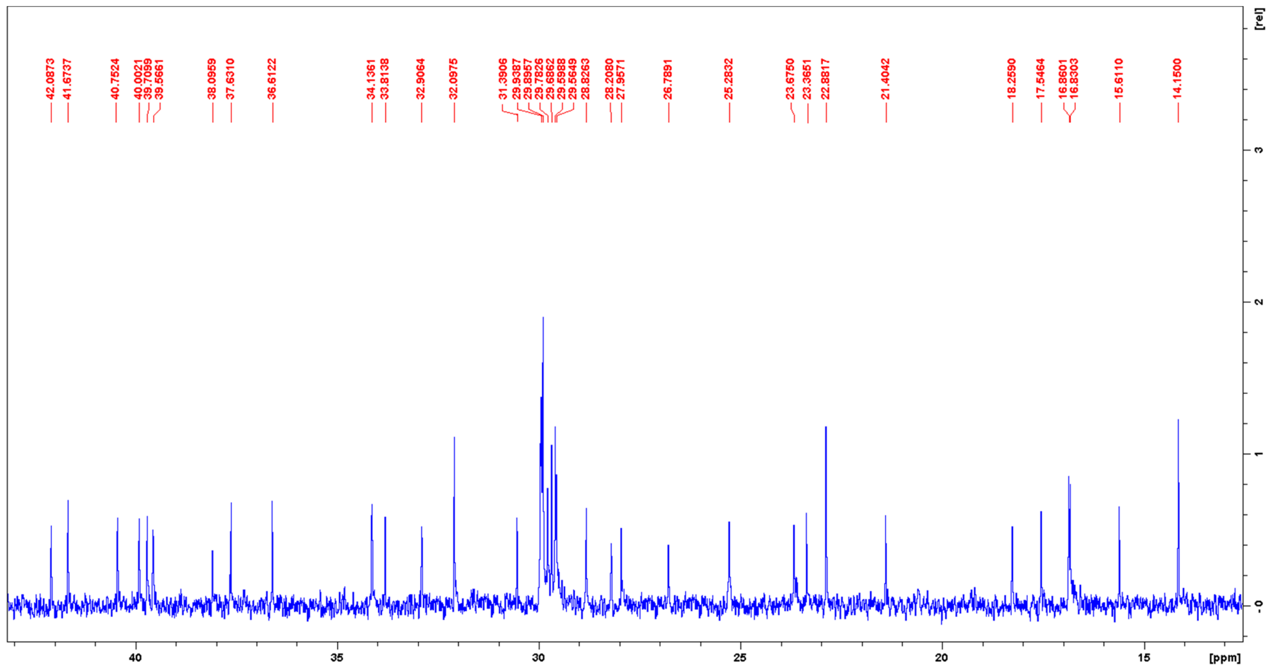

Figure S3. ^{13}CNMR spectrum of **1** (Exp.).

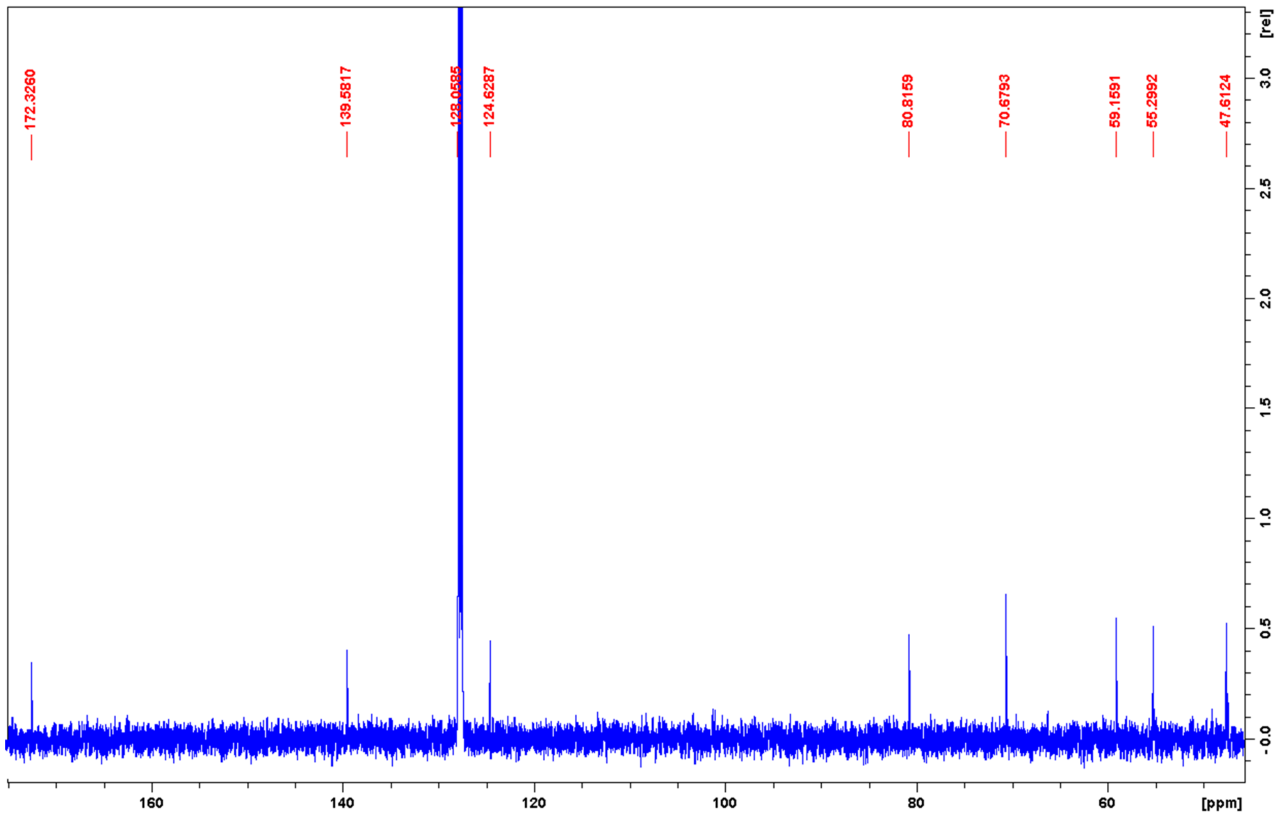

Figure S4. ^{13}CNMR spectrum of **1** (Exp.).

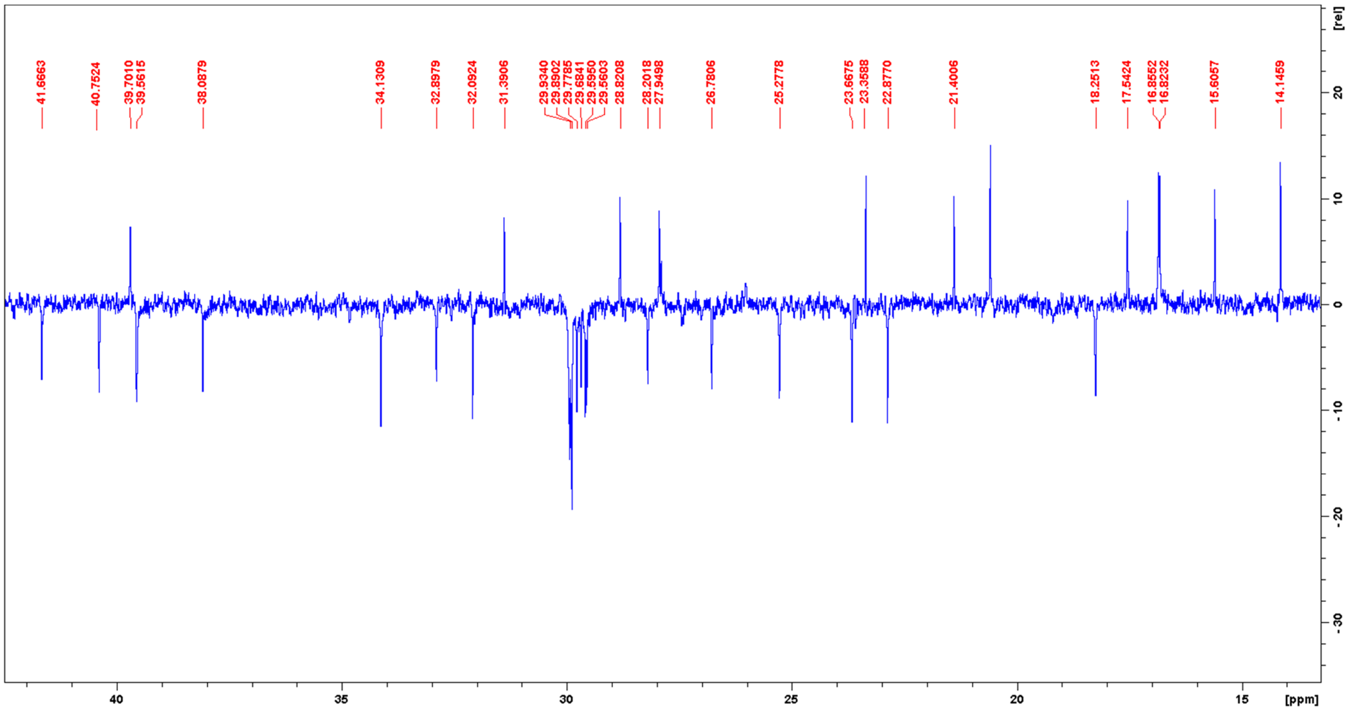

Figure S5. DEPT135 spectrum of **1** (Exp.).

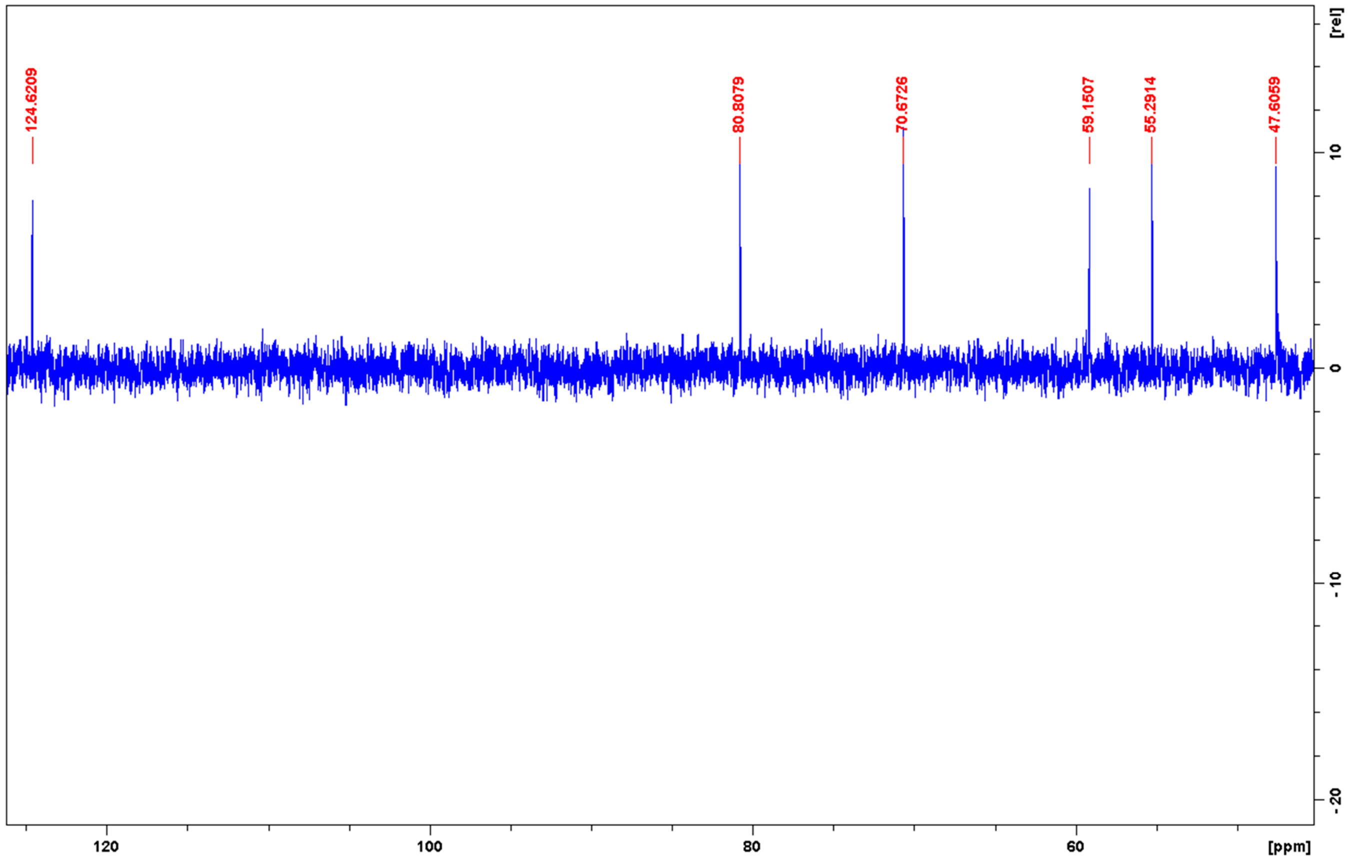

Figure S6. DEPT135 spectrum of **1** (Exp.).

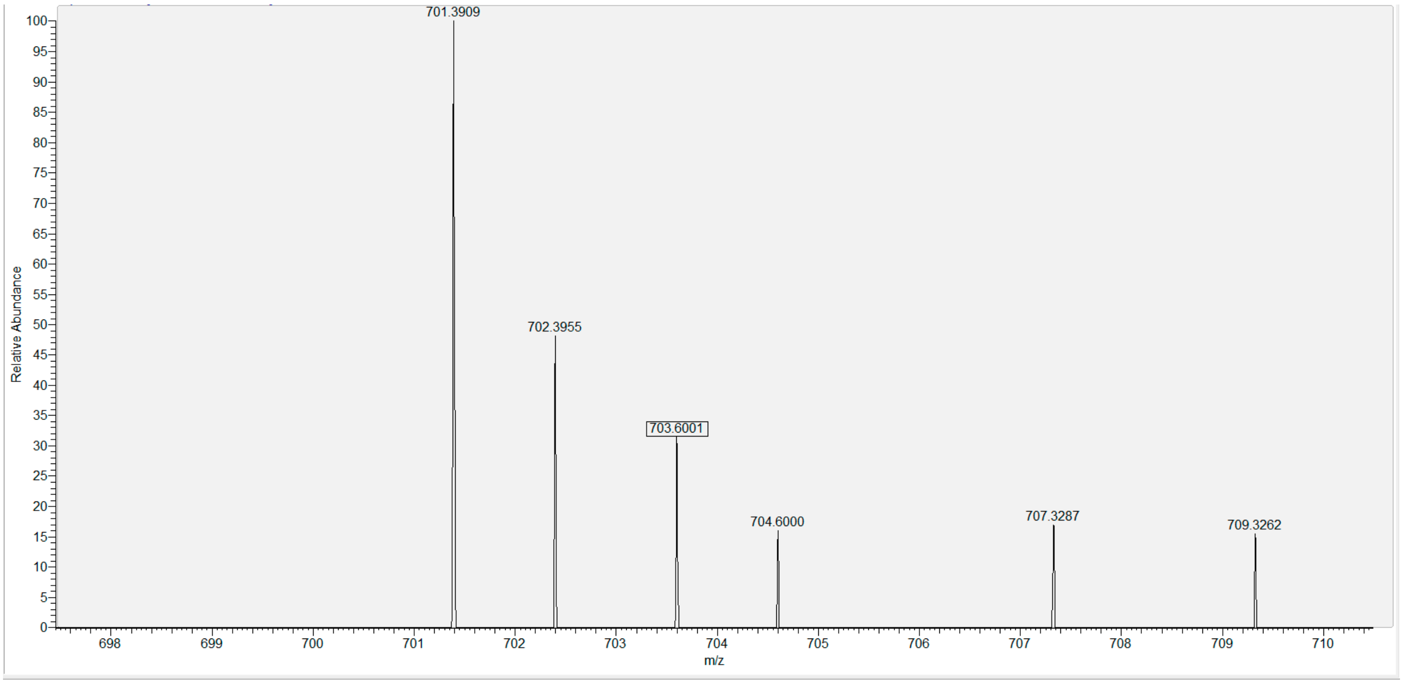

Figure S7. HRESIMS spectrum of **1** (Positive mode).

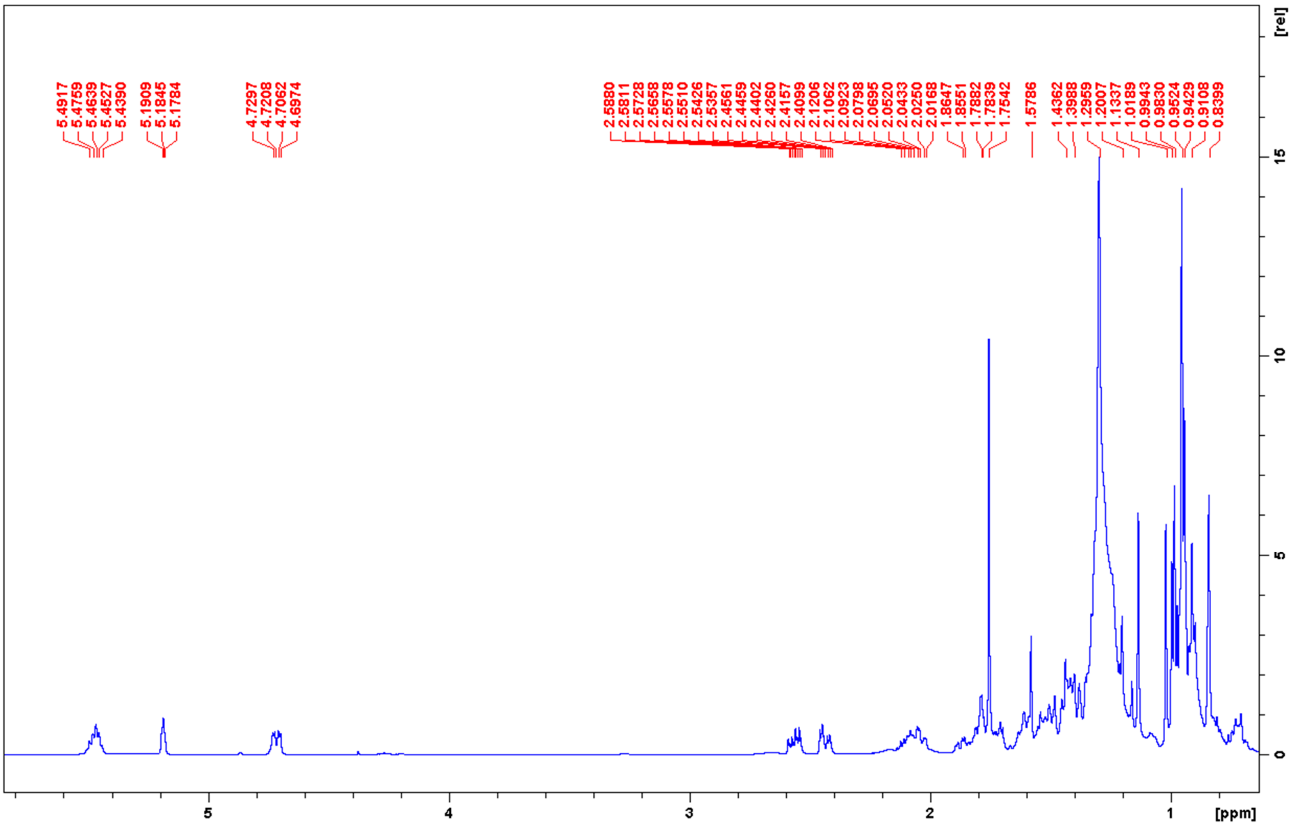

Figure S8. ^{1}HNMR spectrum of **1a**.

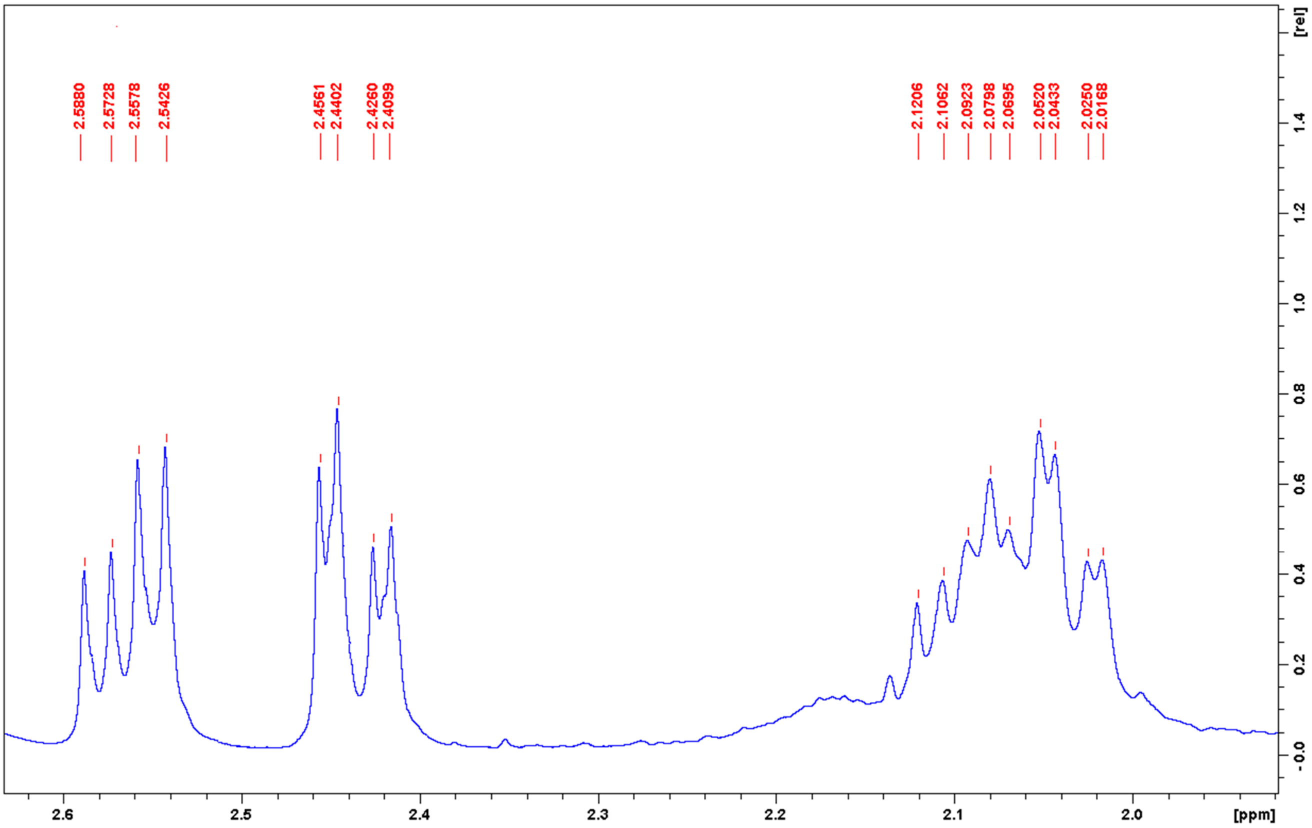

Figure S9. ^{1}HNMR spectrum of **1a** (Exp.).

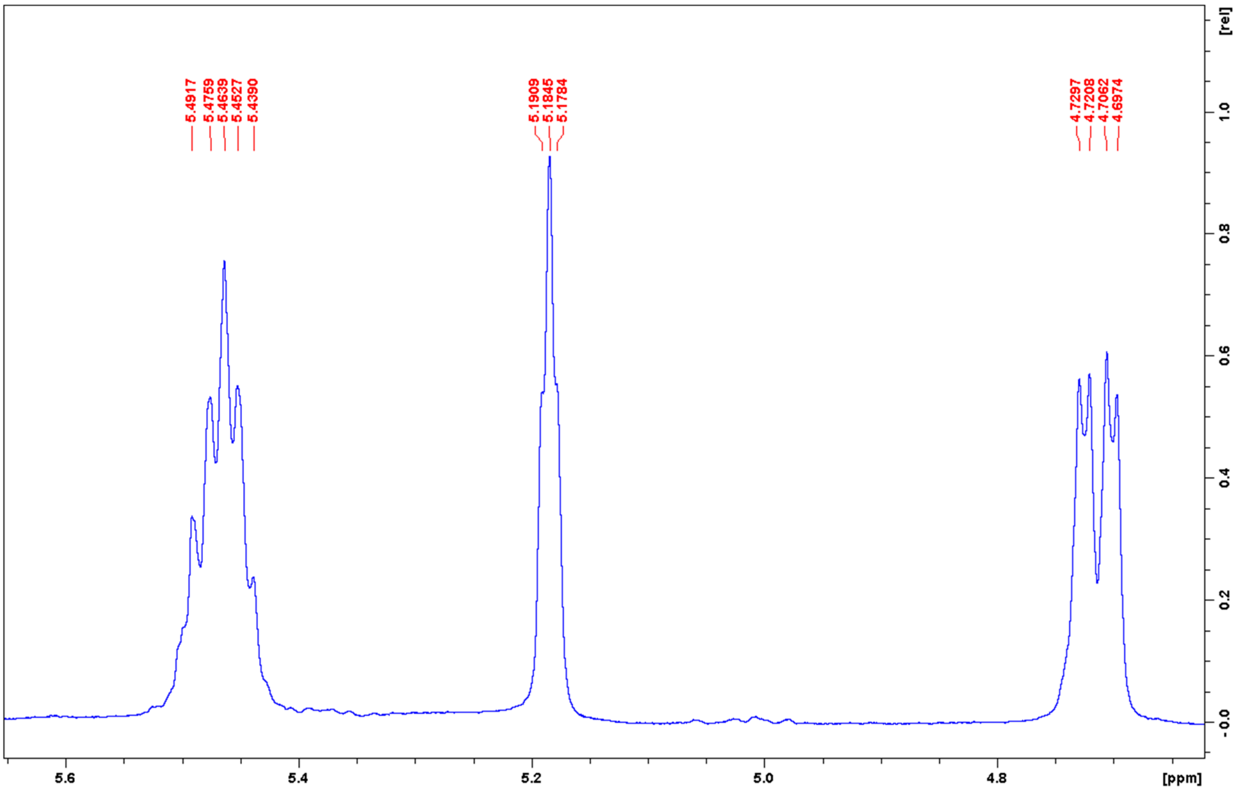

Figure S10. ^{1}HNMR spectrum of **1a** (Exp.).

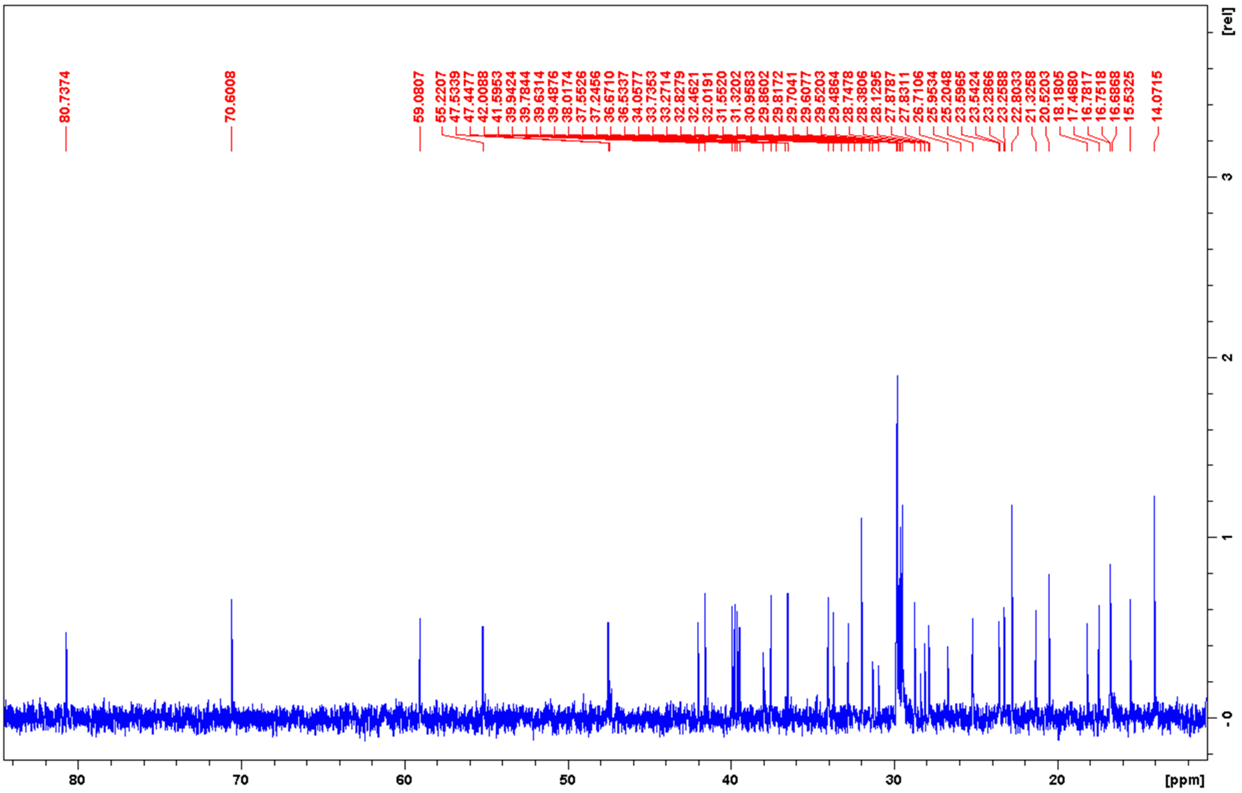

Figure S11. ^{13}CNMR spectrum of **1a** (Exp.).

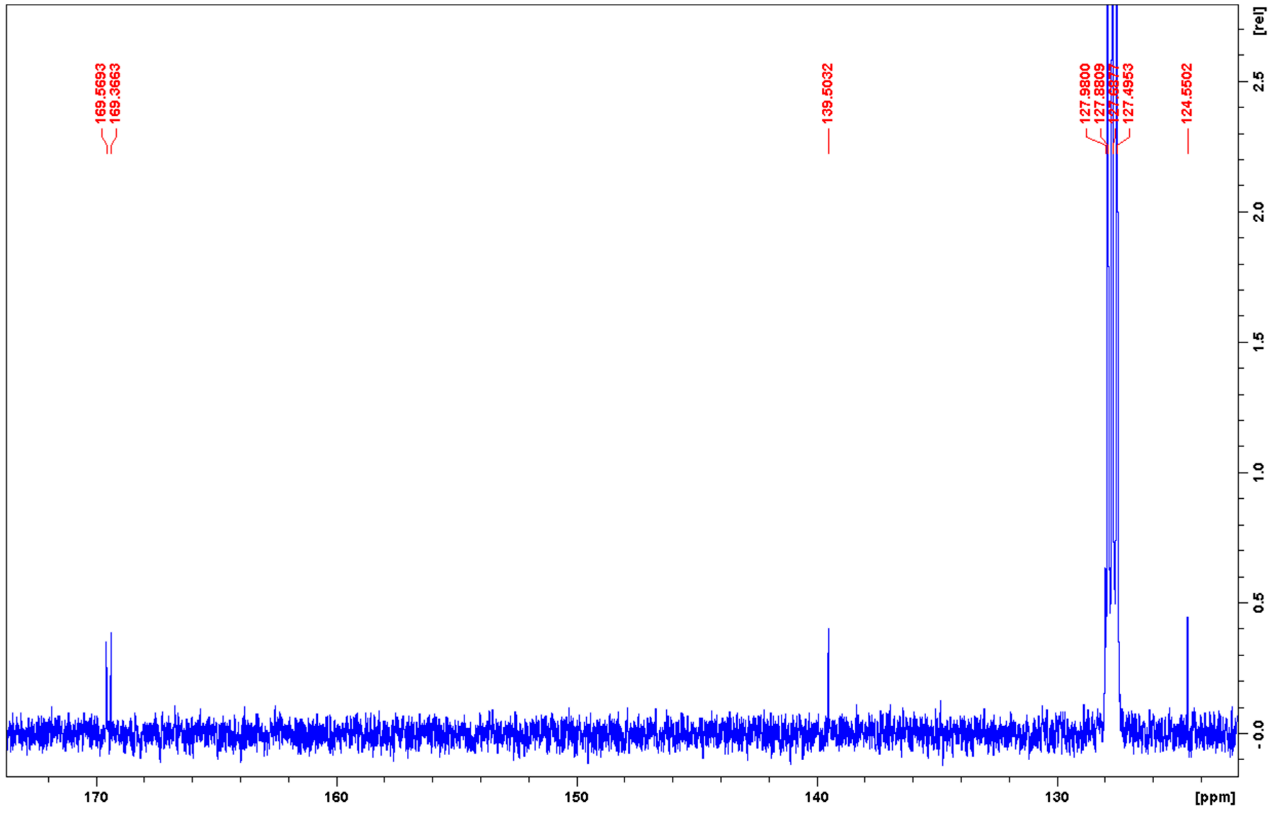

Figure S12. ^{13}CNMR spectrum of **1a** (Exp.).

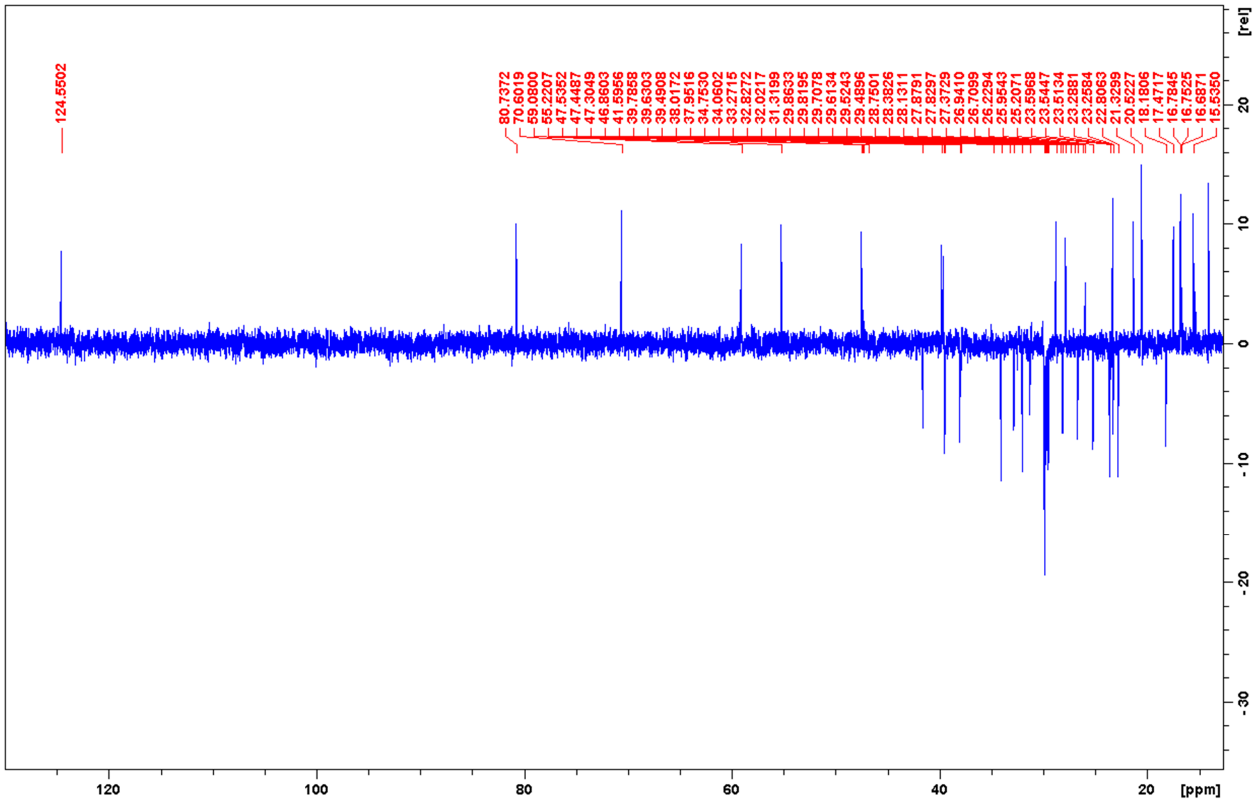

Figure S13. DEPT135 spectrum of **1a**.

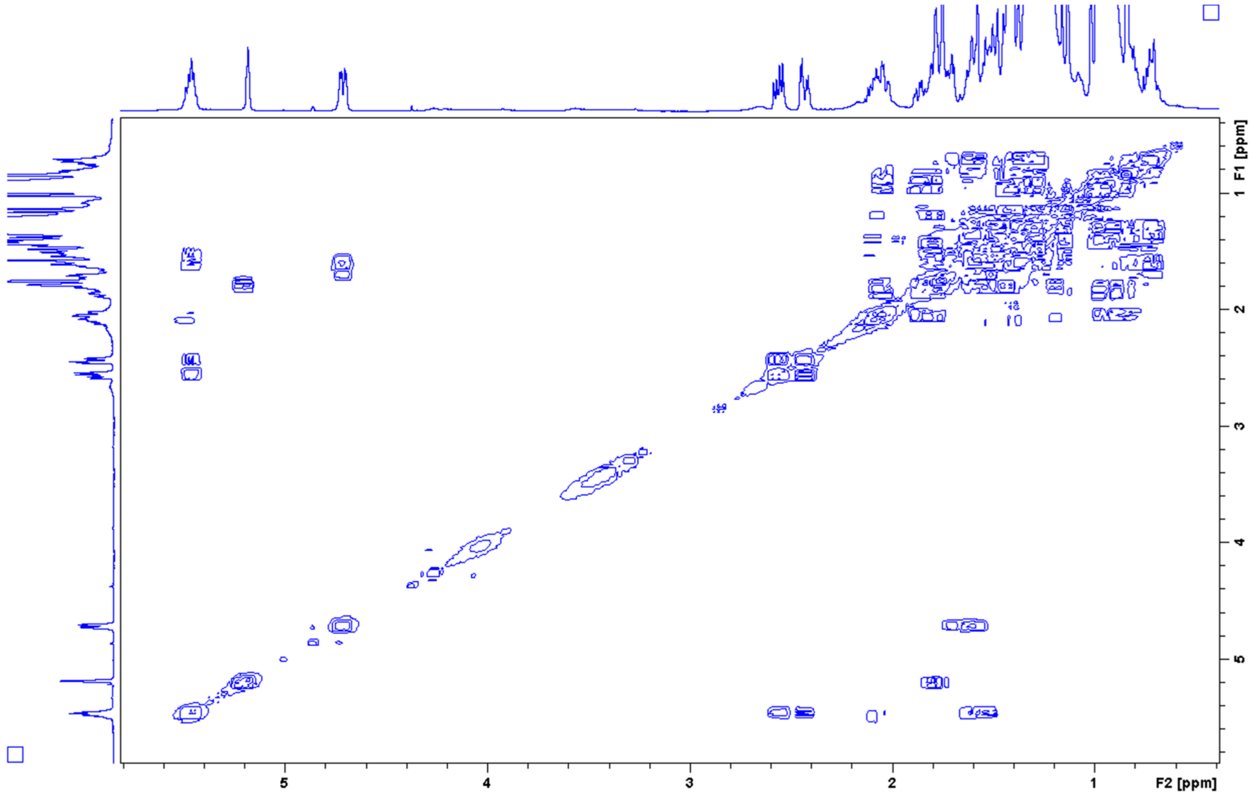

Figure S14. COSY spectrum of **1a**.

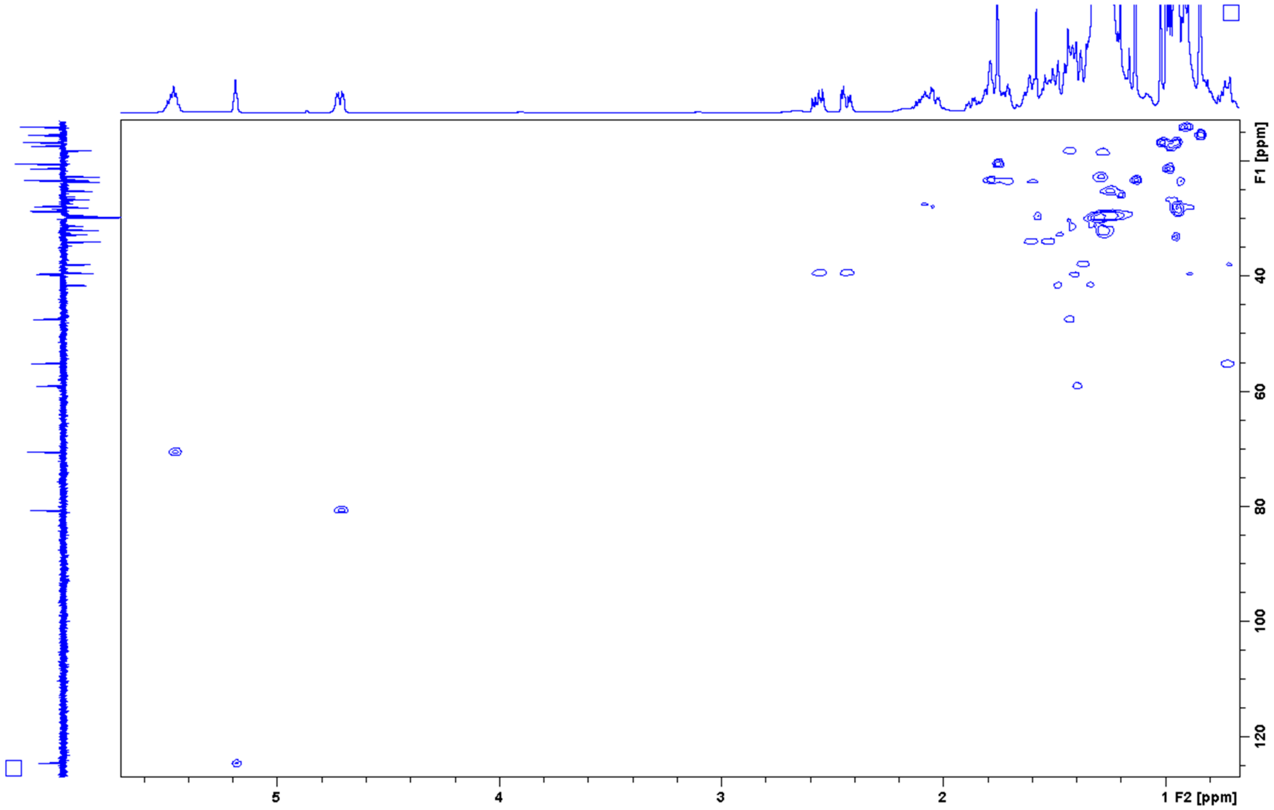

Figure S15. HSQC spectrum of **1a**.

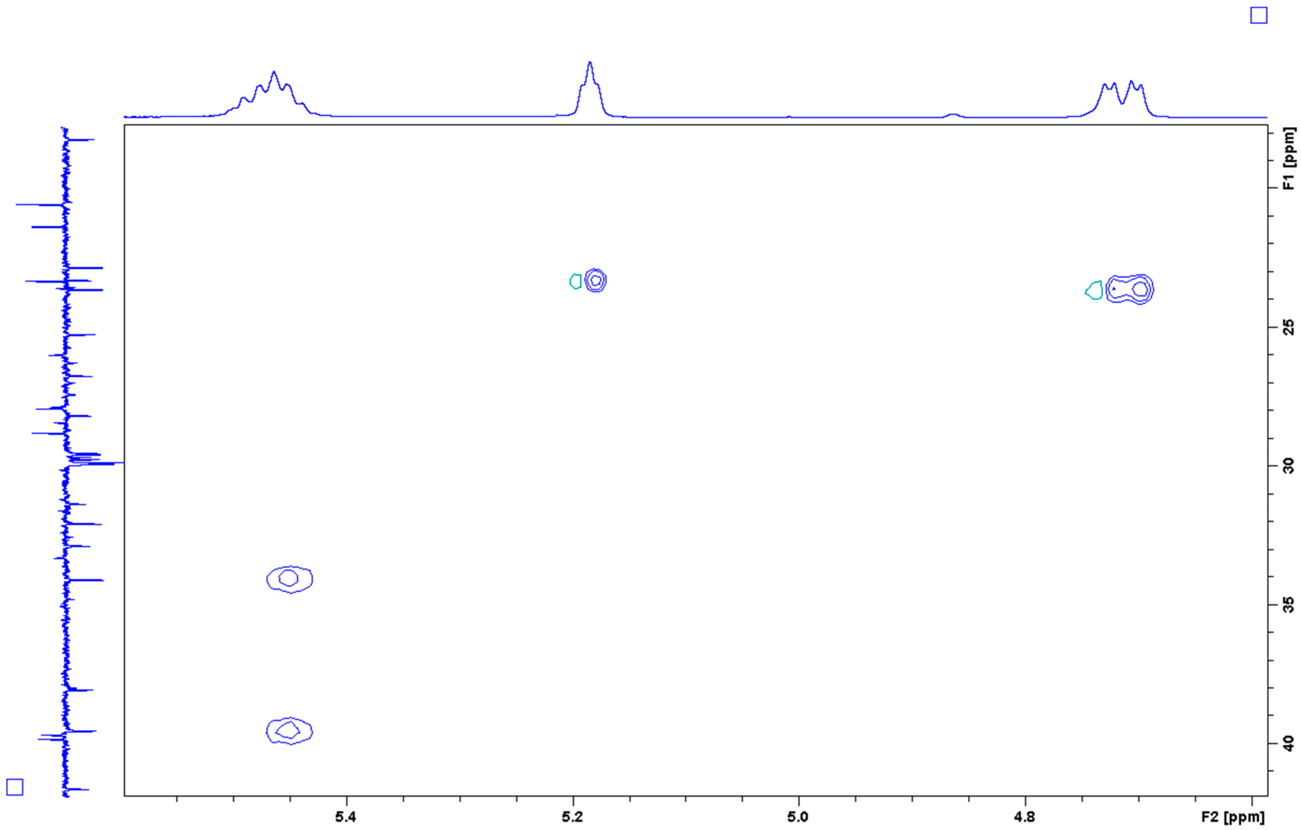

Figure S16. H2BC spectrum of **1a** (Exp.).

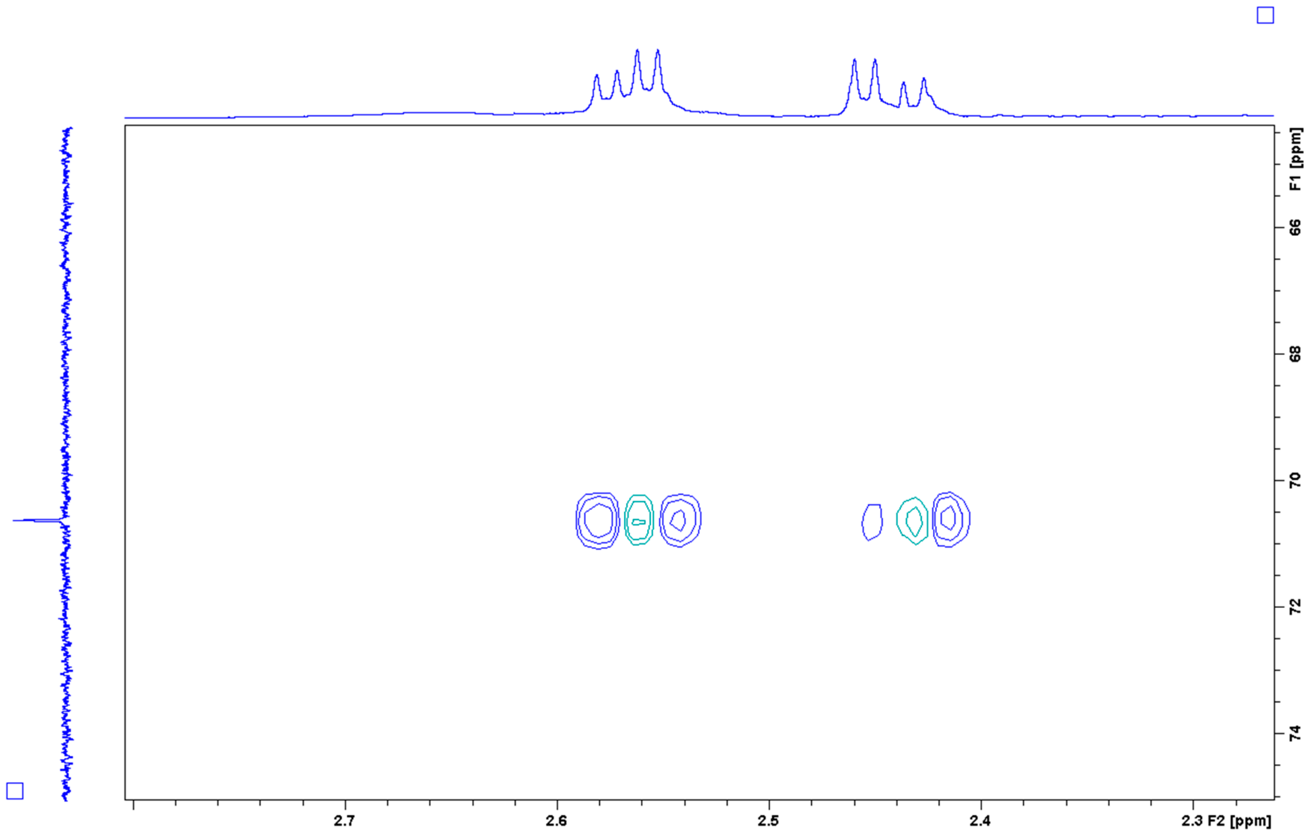

Figure S17. H2BC spectrum of **1a** (Exp.).

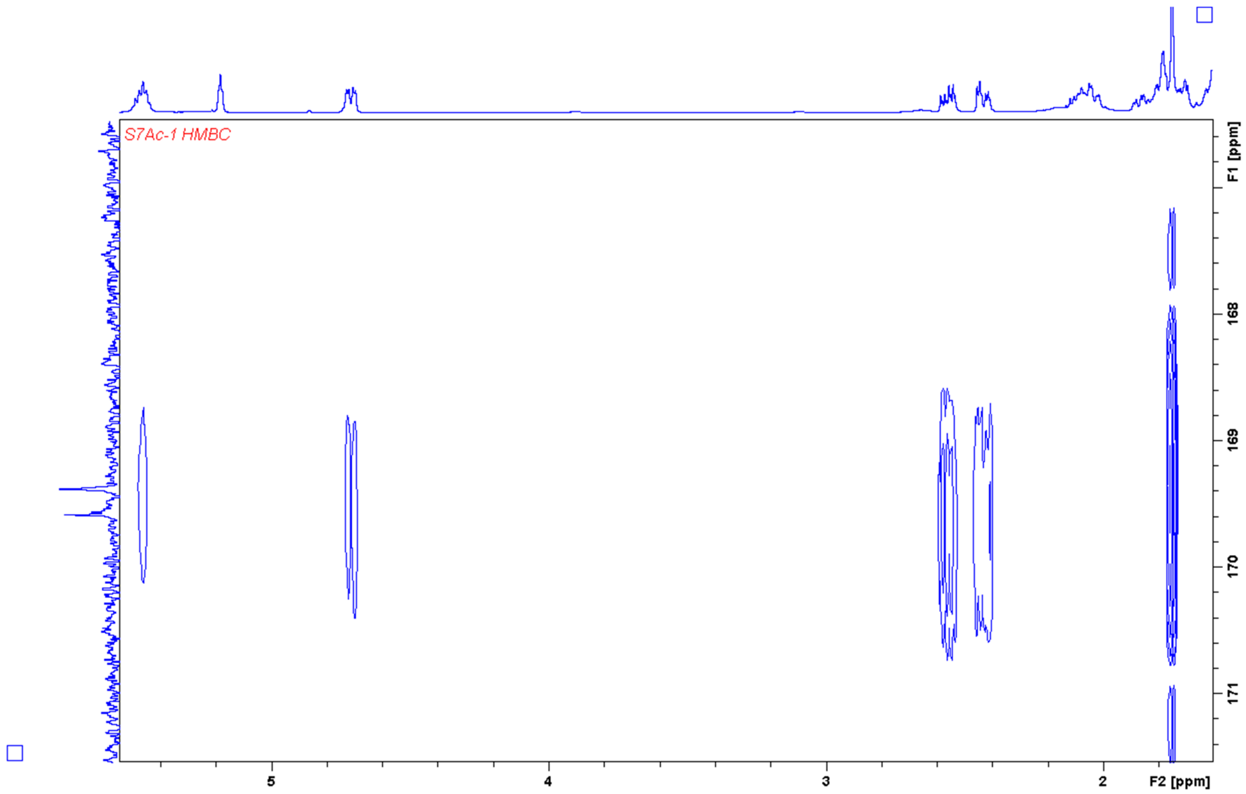

Figure S18. HMBC spectrum of **1a** (Exp.).

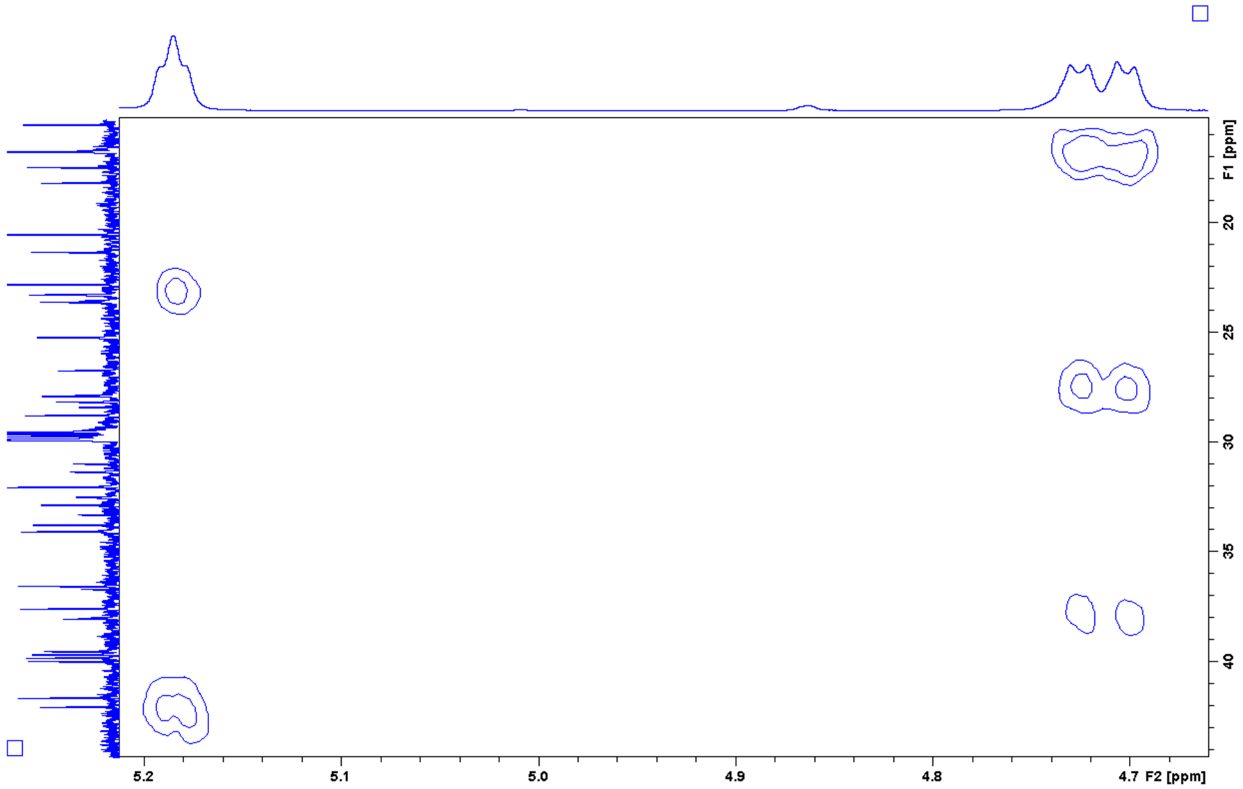

Figure S19. HMBC spectrum of **1a** (Exp.).

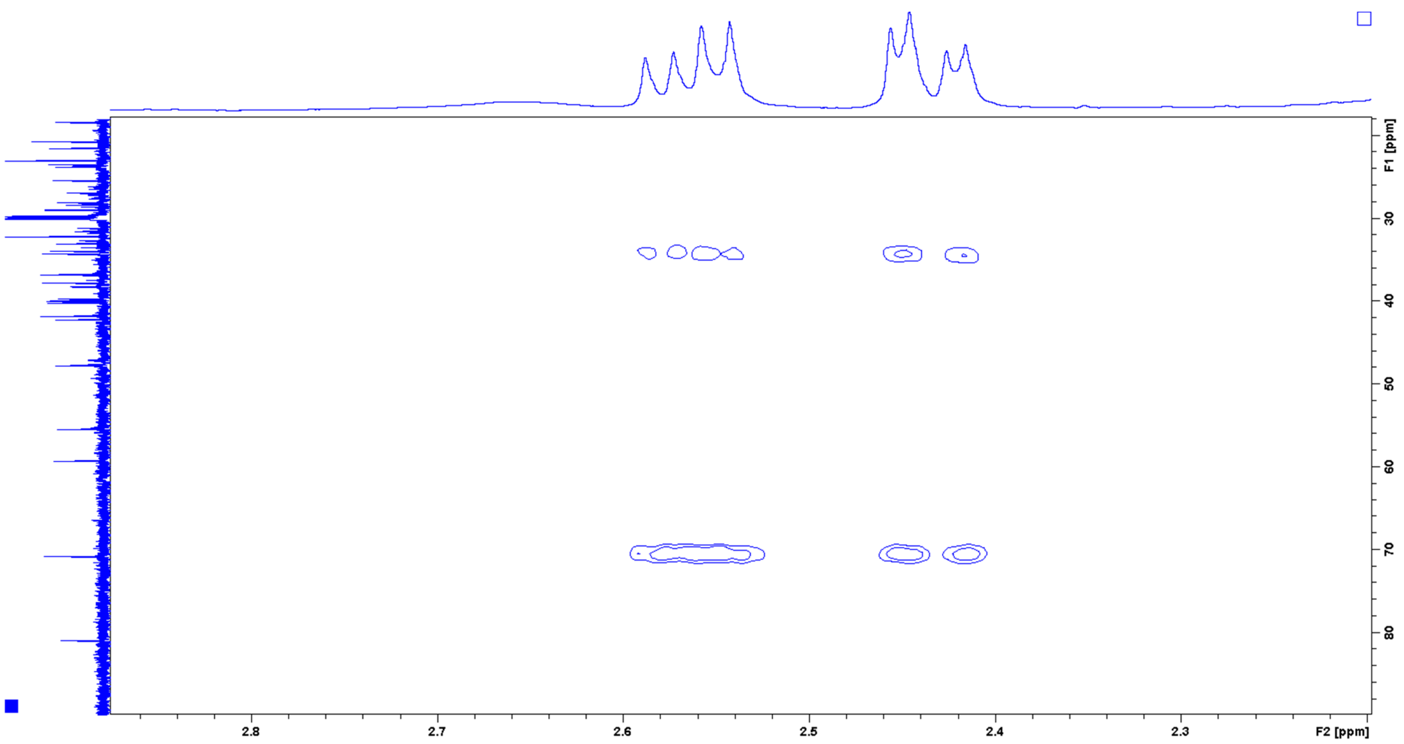

Figure S20. HMBC spectrum of **1a** (Exp.).

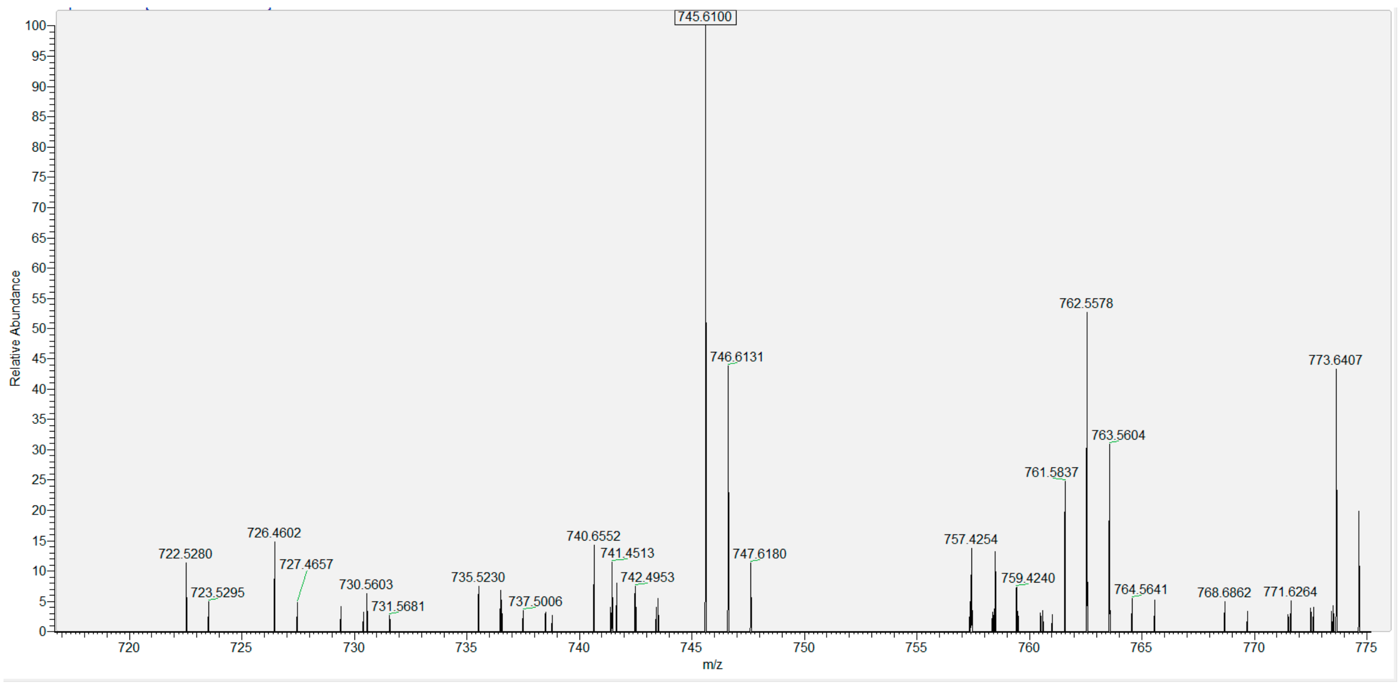

Figure S21. HRESIMS spectrum of **1**a (Positive mode).

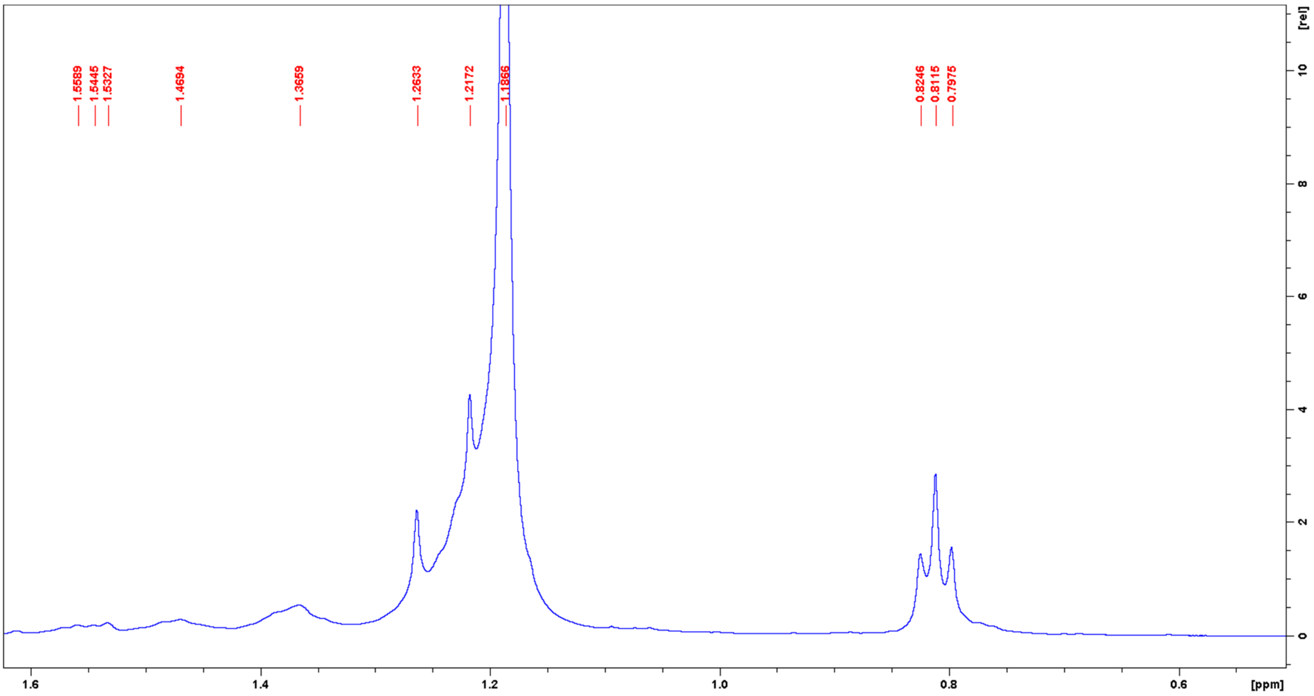

Figure S22. ^{1}HNMR spectrum of **1b** (Exp.).

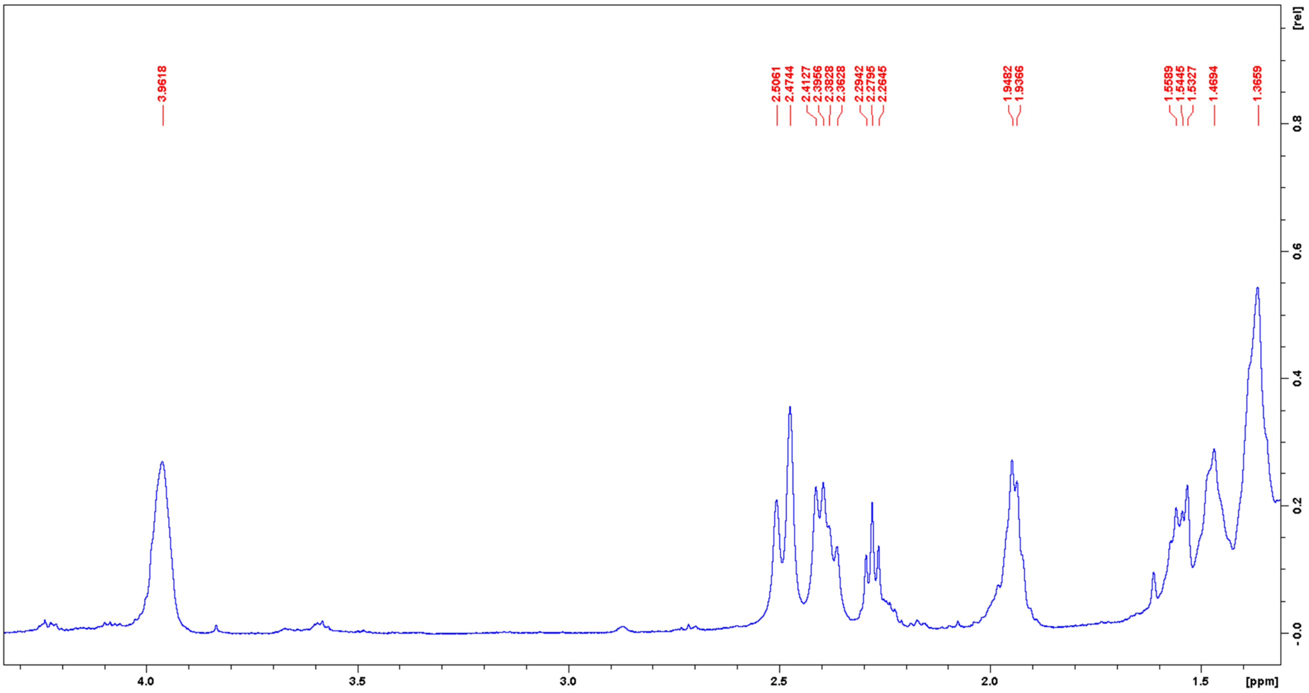

Figure S23. ^{1}HNMR spectrum of **1b** (Exp.).

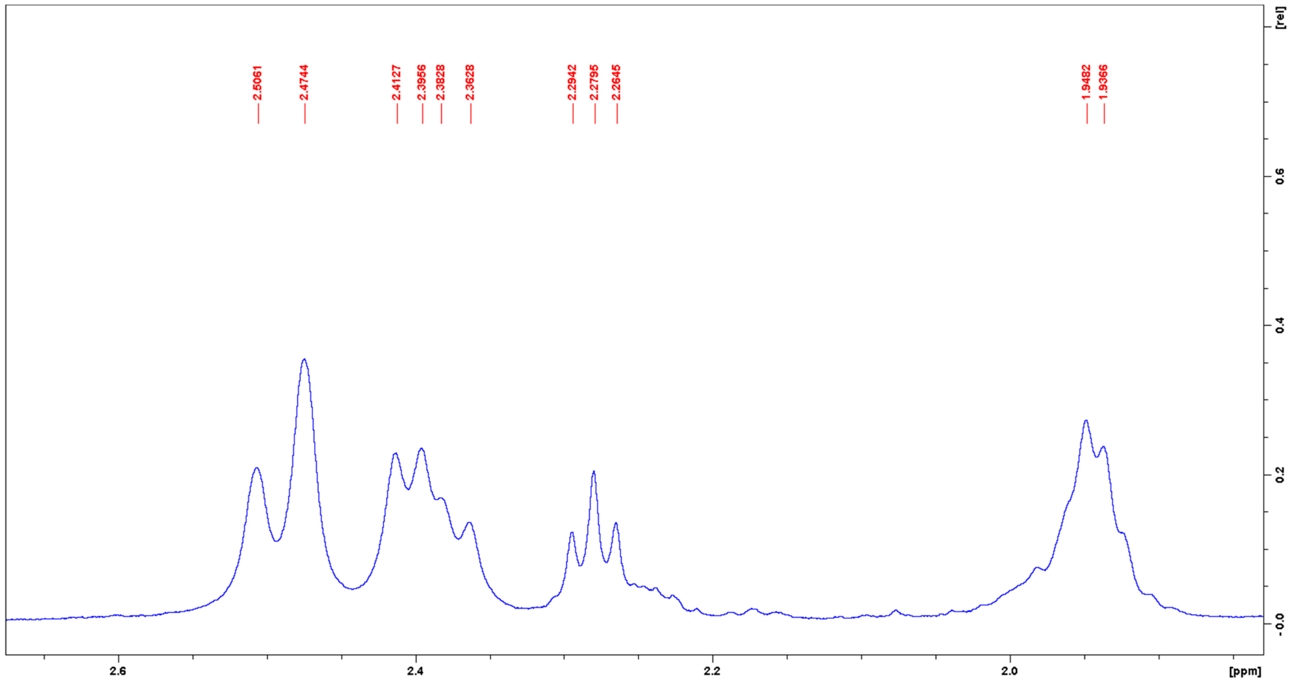

Figure S24. ^{1}HNMR spectrum of **1b** (Exp.).

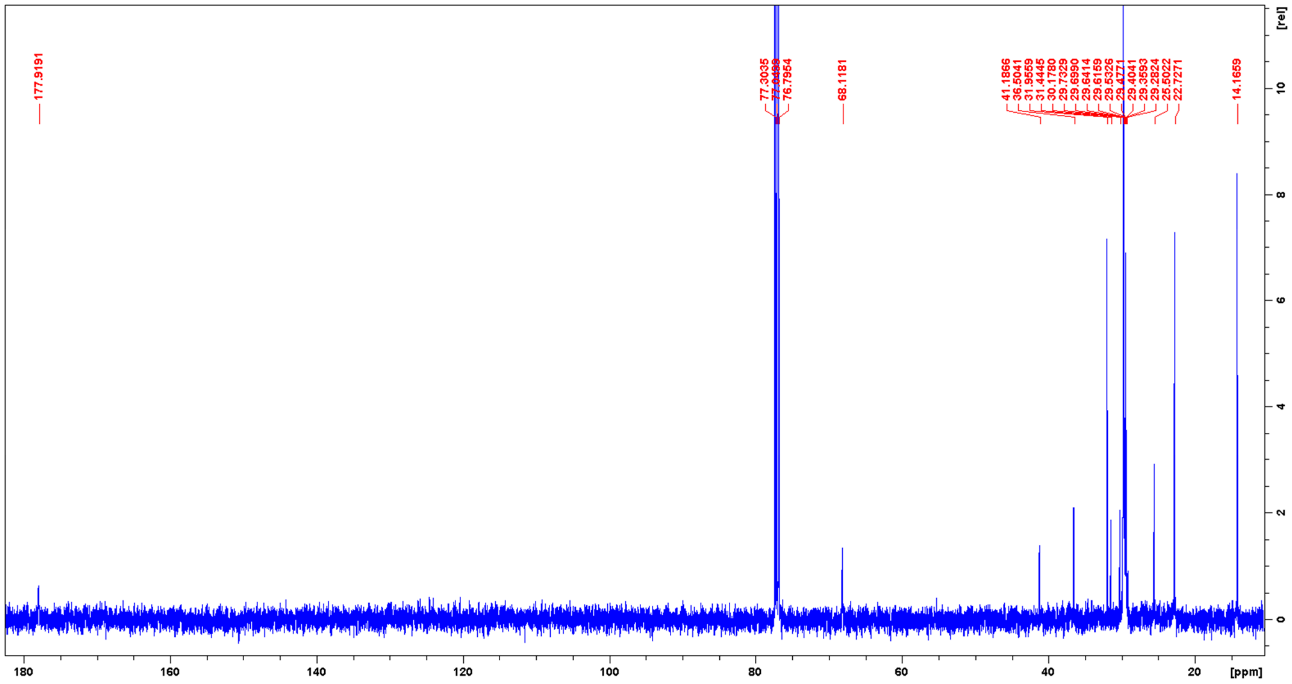

Figure S25. ^{13}CNMR spectrum of **1b**.

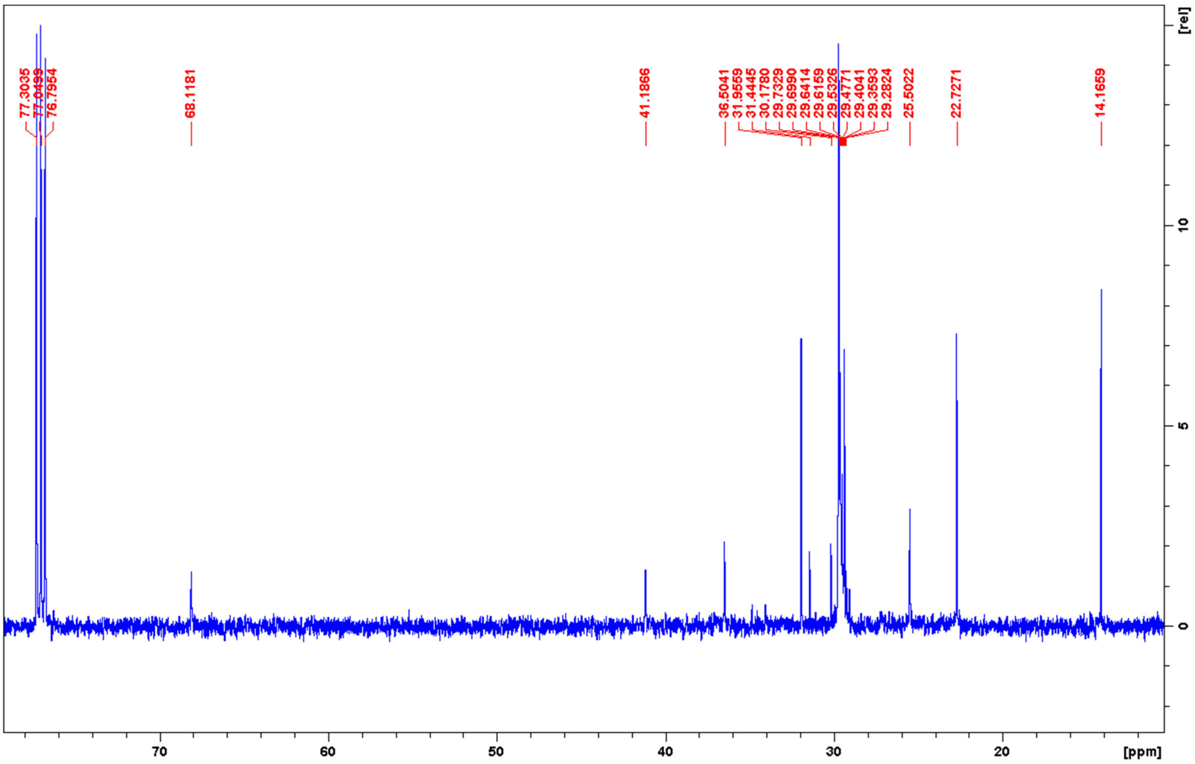

Figure S26. ^{13}CNMR spectrum of **1b** (Exp.).

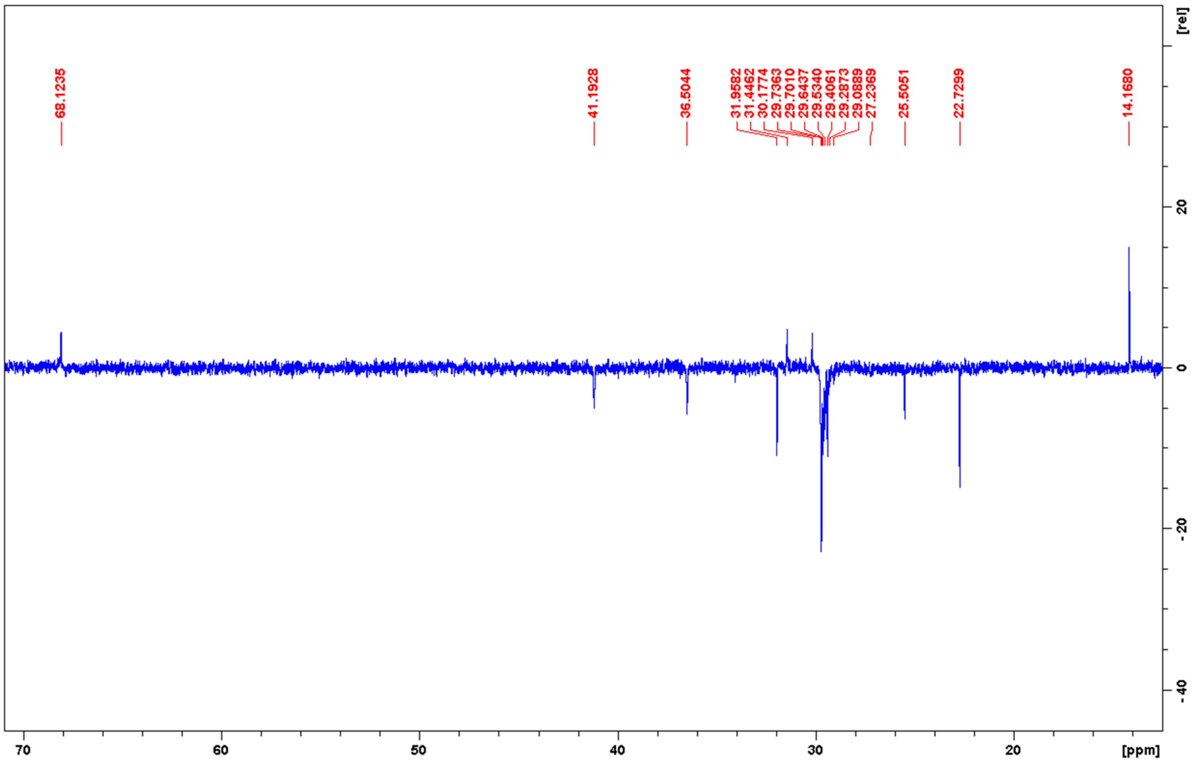

Figure S27. DEPT135 spectrum of **1b**.

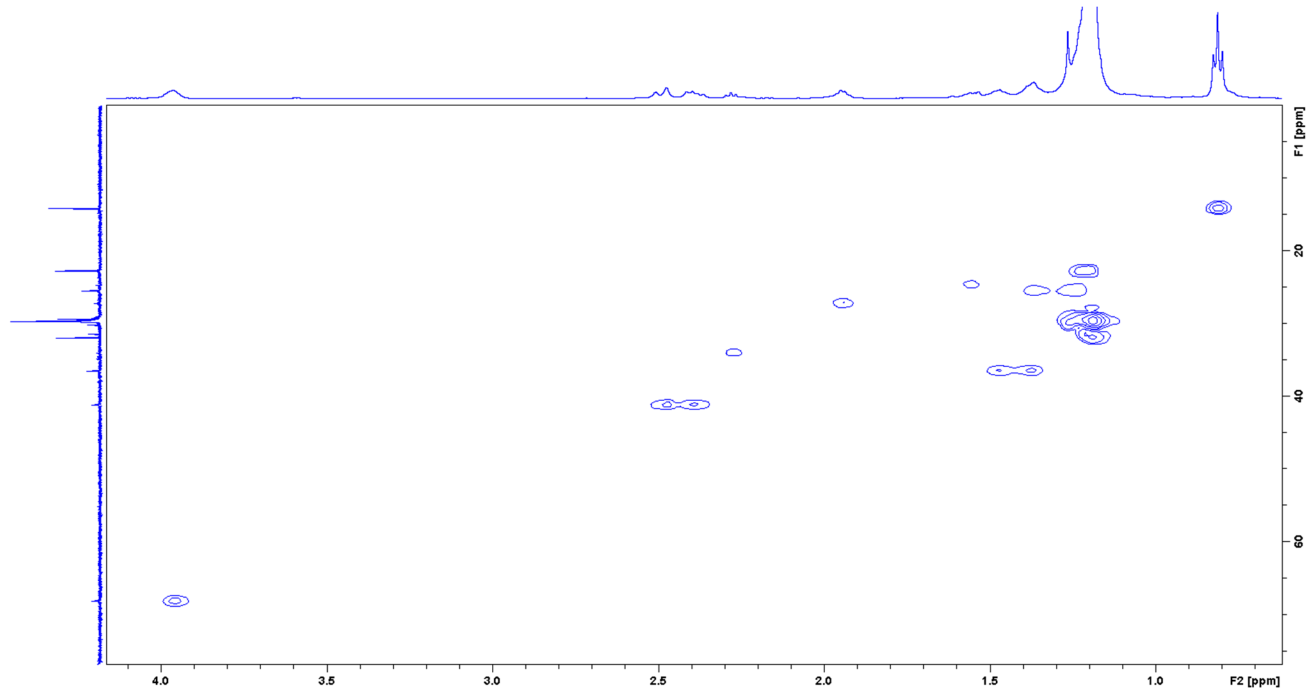

Figure S28. HSQC spectrum of **1b**.

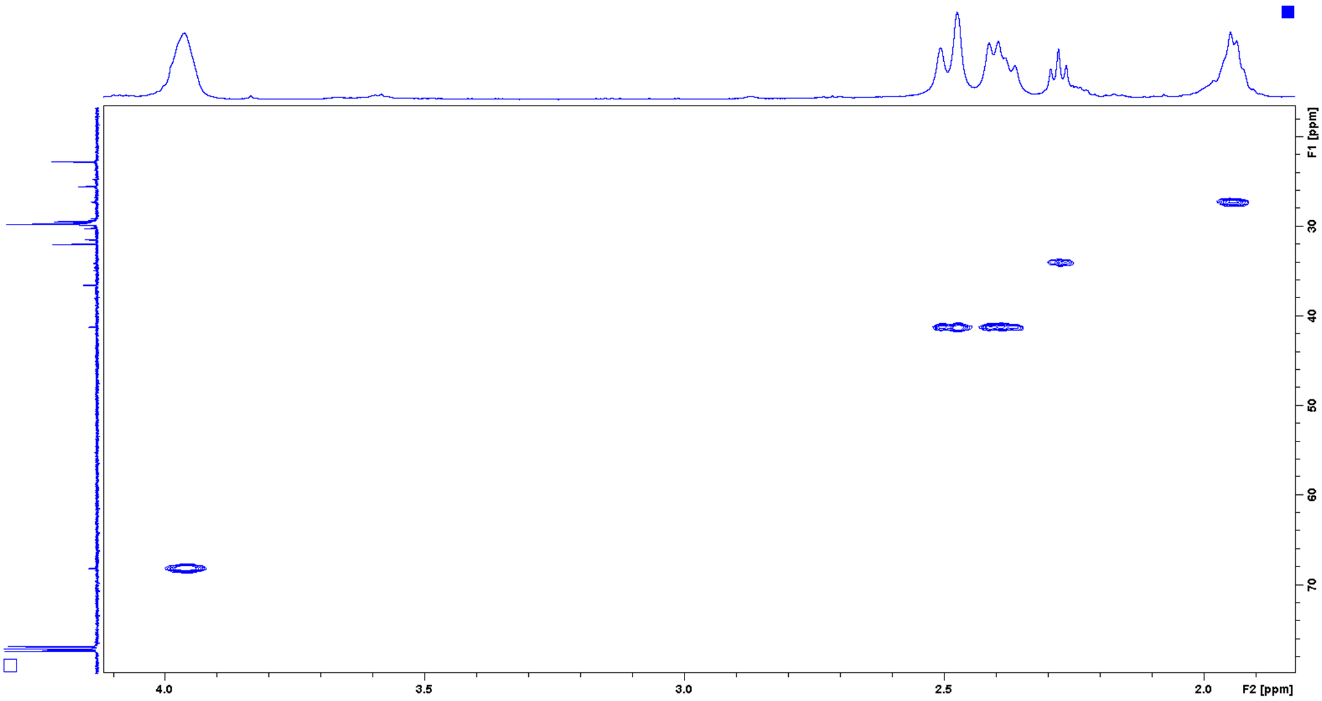

Figure S29. HSQC spectrum of **1b** (Exp.).

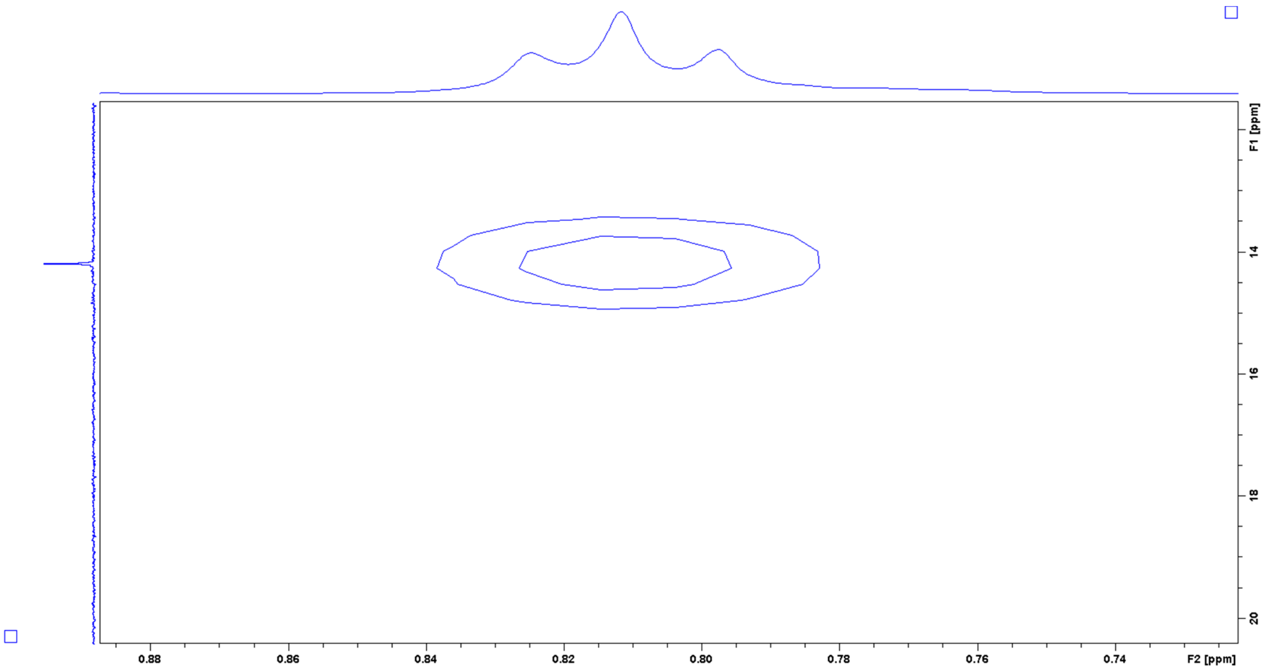

Figure S30. HSQC spectrum of **1b** (Exp.).

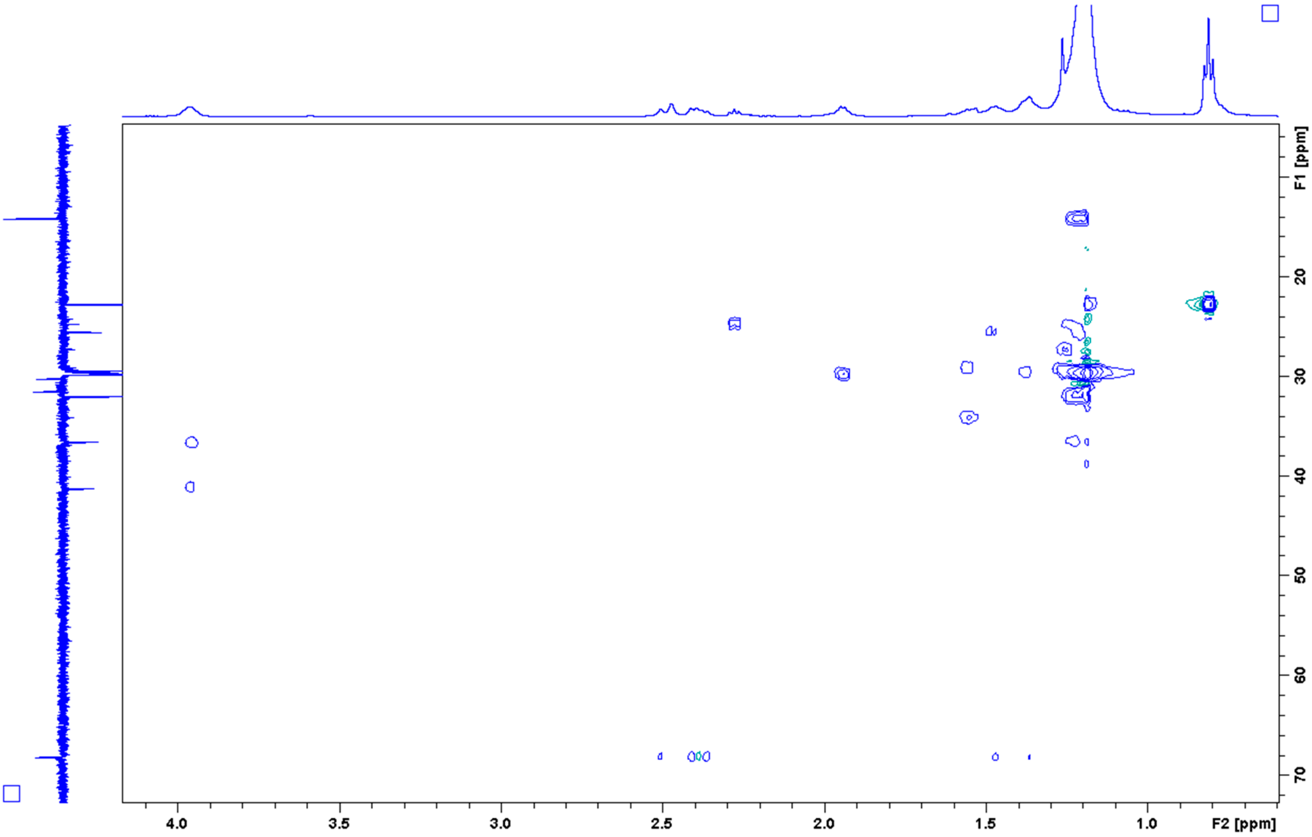

Figure S31. H2BC spectrum of **1b**.

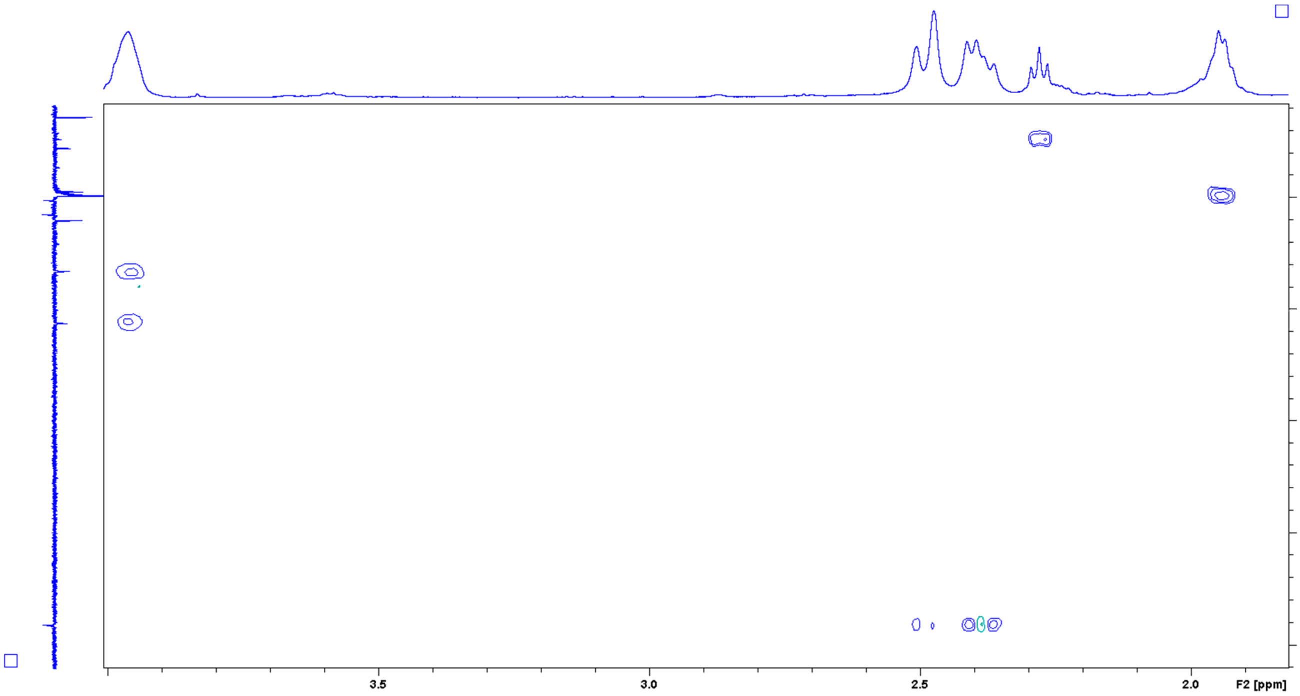

Figure S32. H2BC spectrum of **1b** (Exp.).

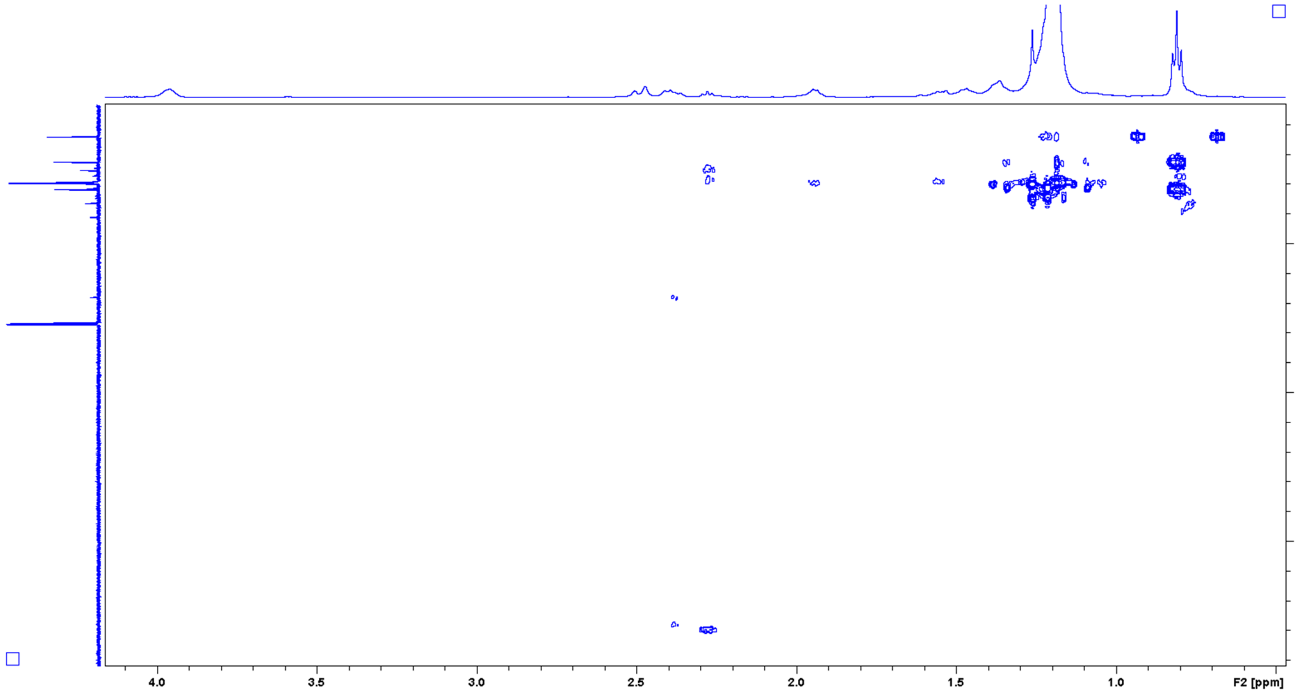

Figure S33. HMBC spectrum of **1b**.

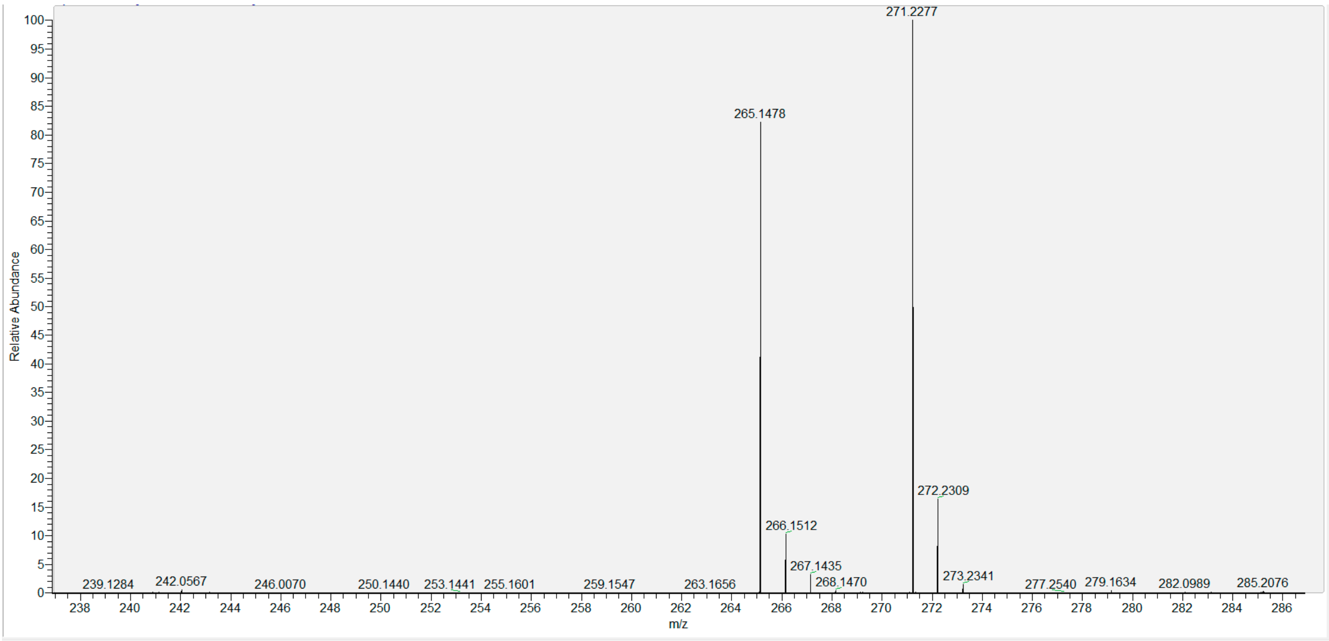

Figure S34. HRESIMS spectrum of **1b** (Negative mode).

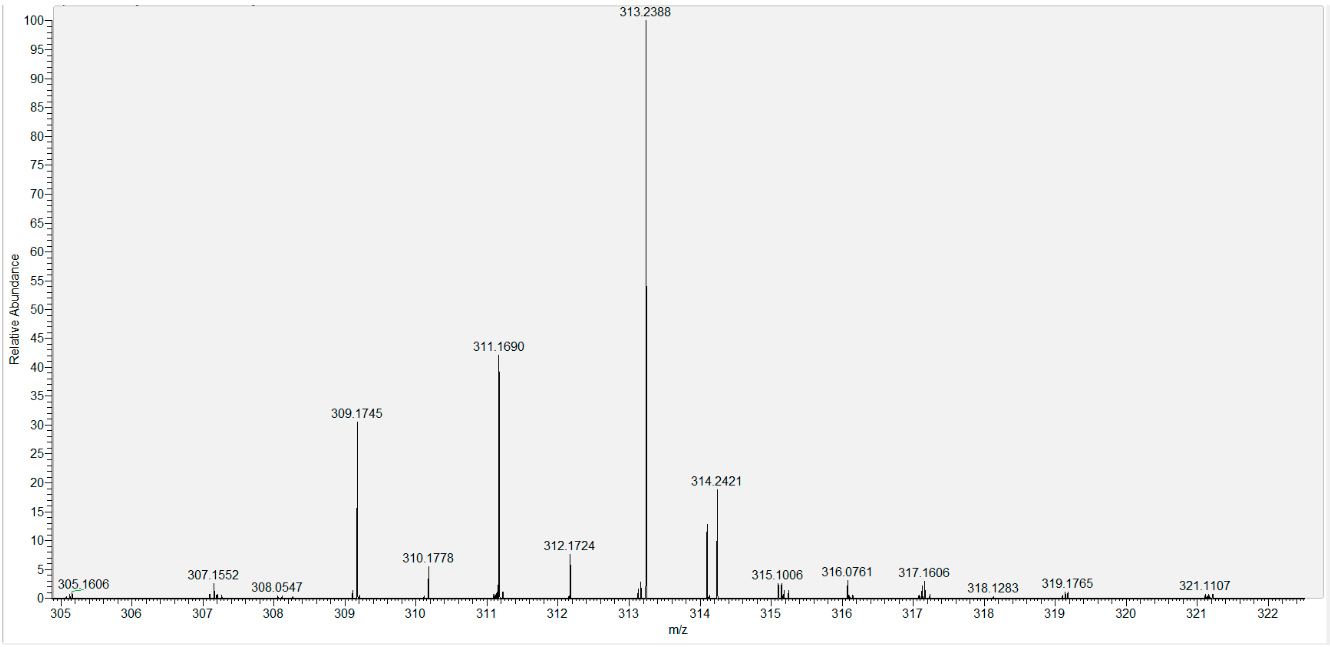

Figure S35. HRESIMS spectrum of **1b Ac** (Negative mode).

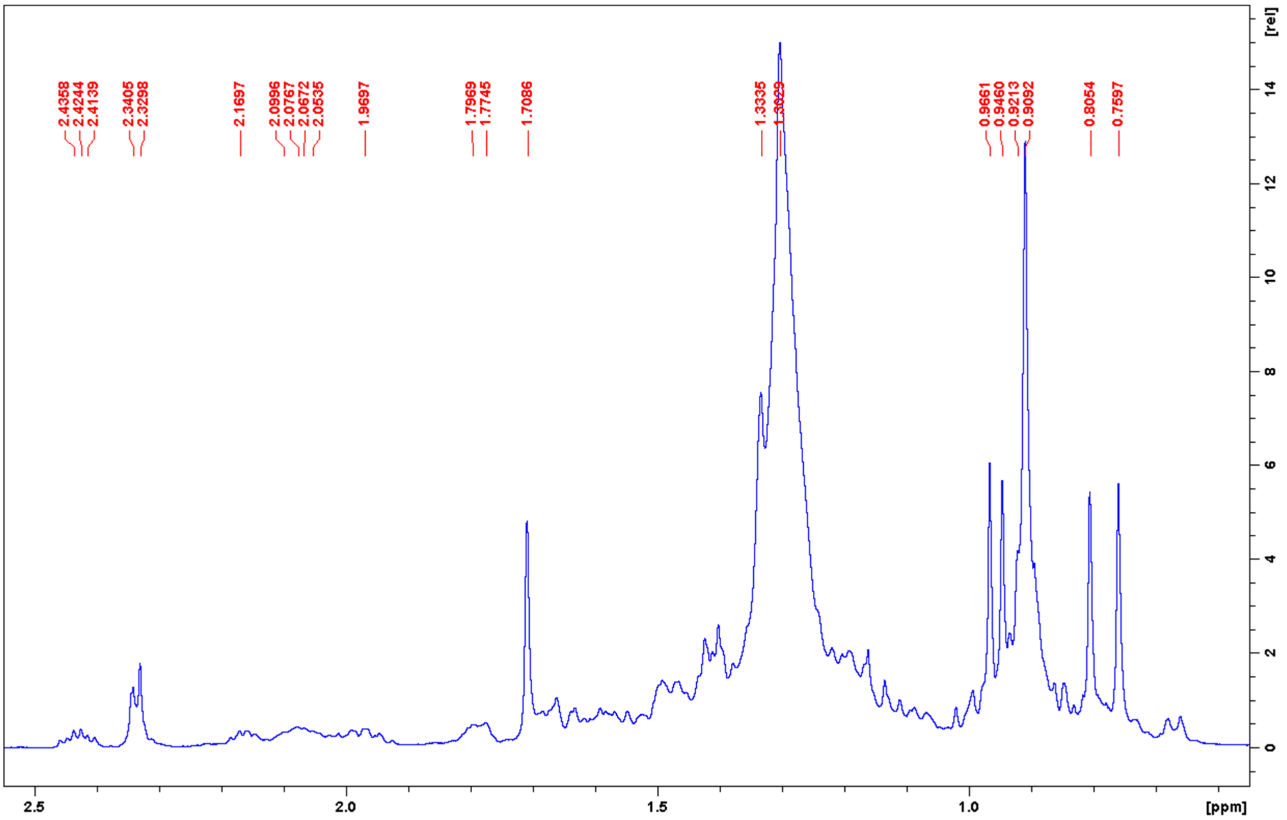

Figure S36. ^{1}HNMR spectrum of **2** (Exp.).

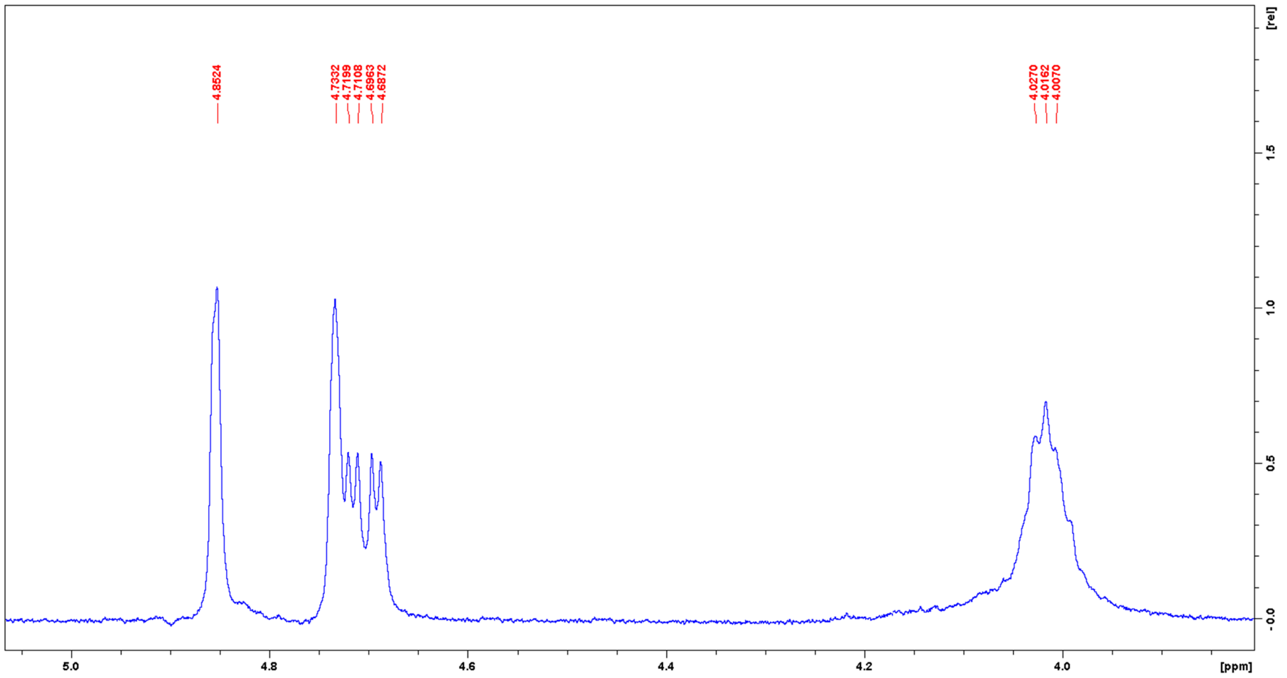

Figure S37. ^{1}HNMR spectrum of **2** (Exp.).

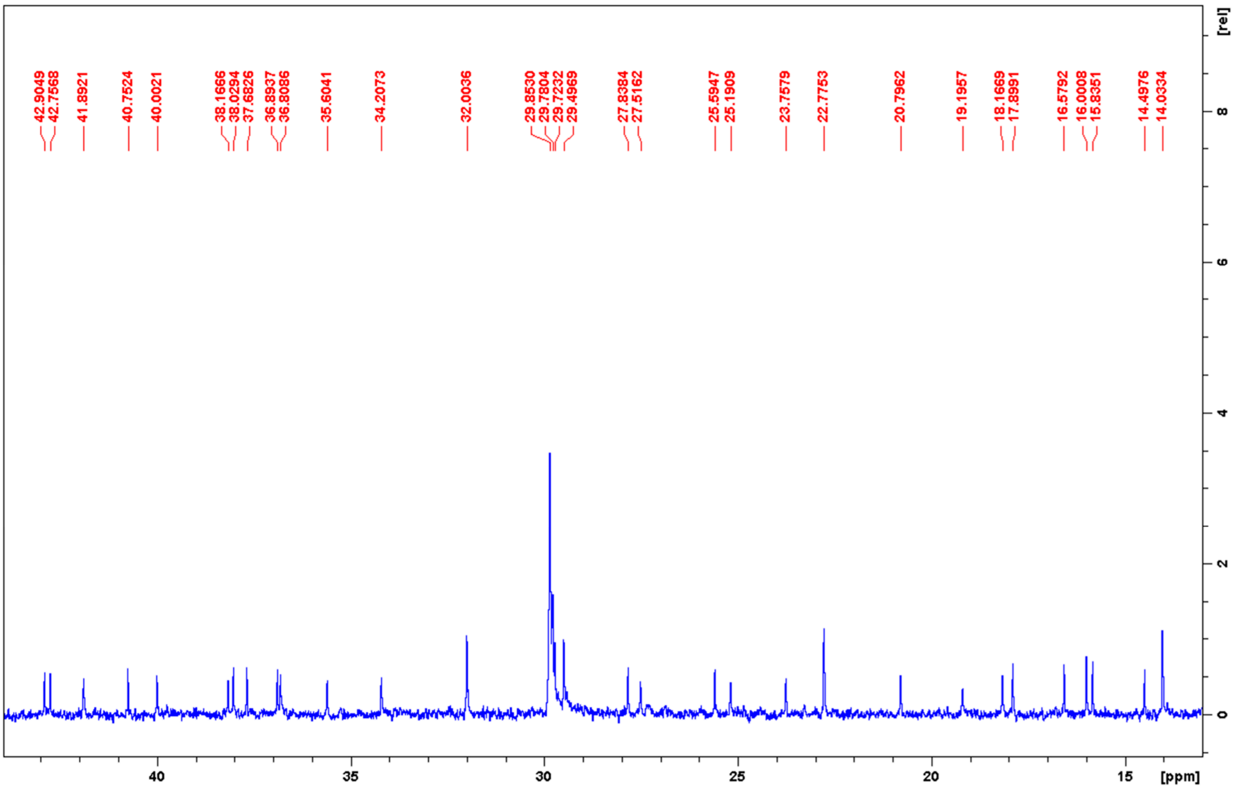

Figure S38. ^{13}CNMR spectrum of **2** (Exp.).

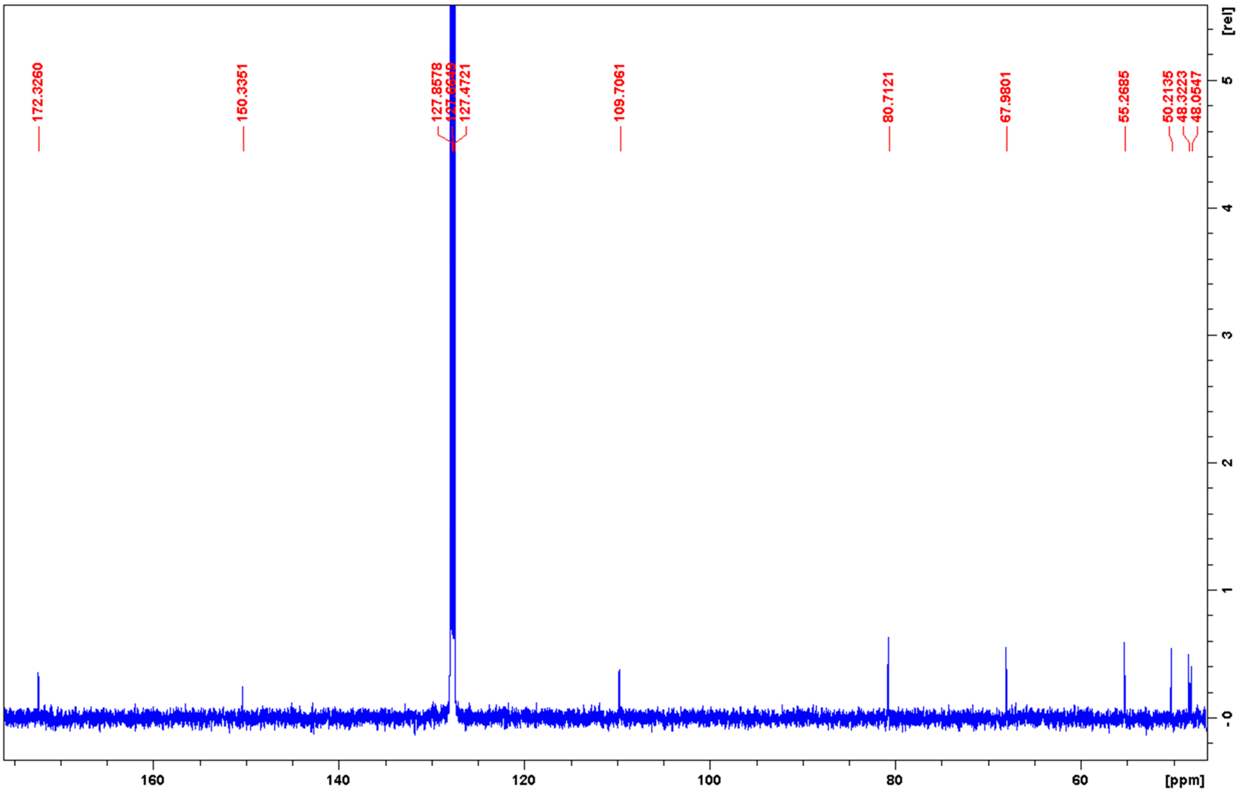

Figure S39. ^{13}CNMR spectrum of **2** (Exp.).

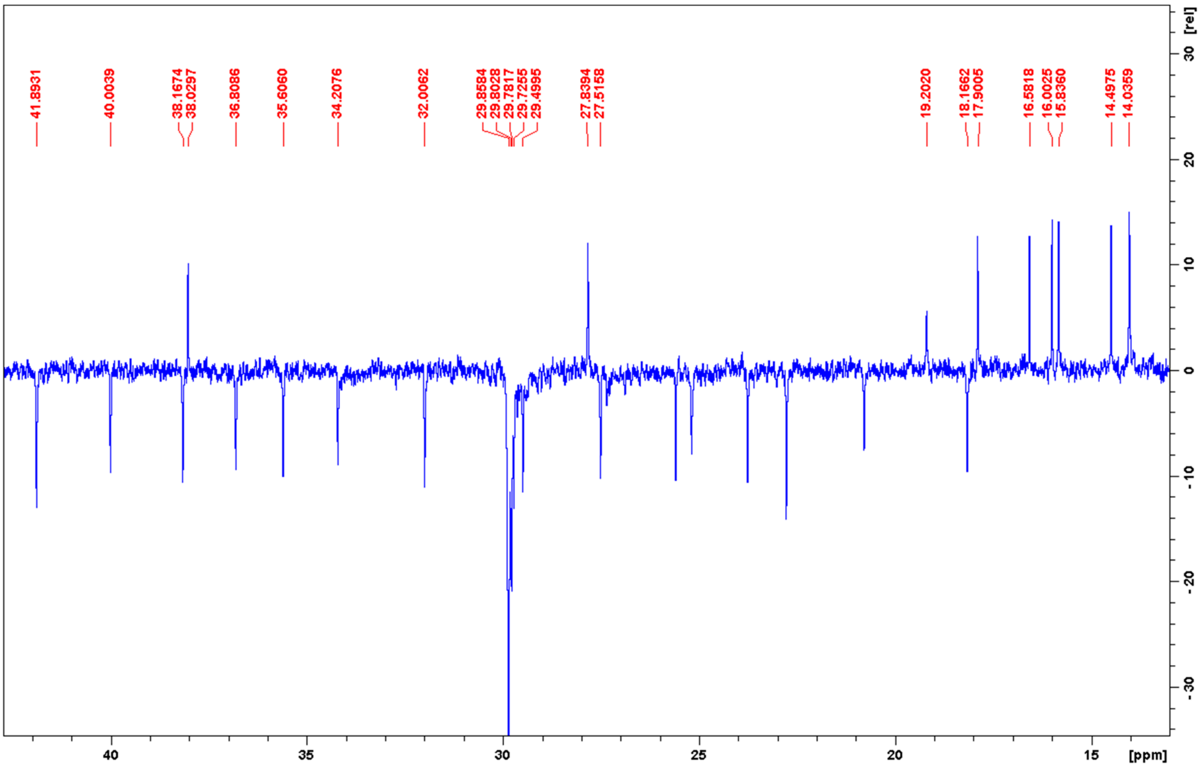

Figure S40. DEPT135 spectrum of **2** (Exp.).

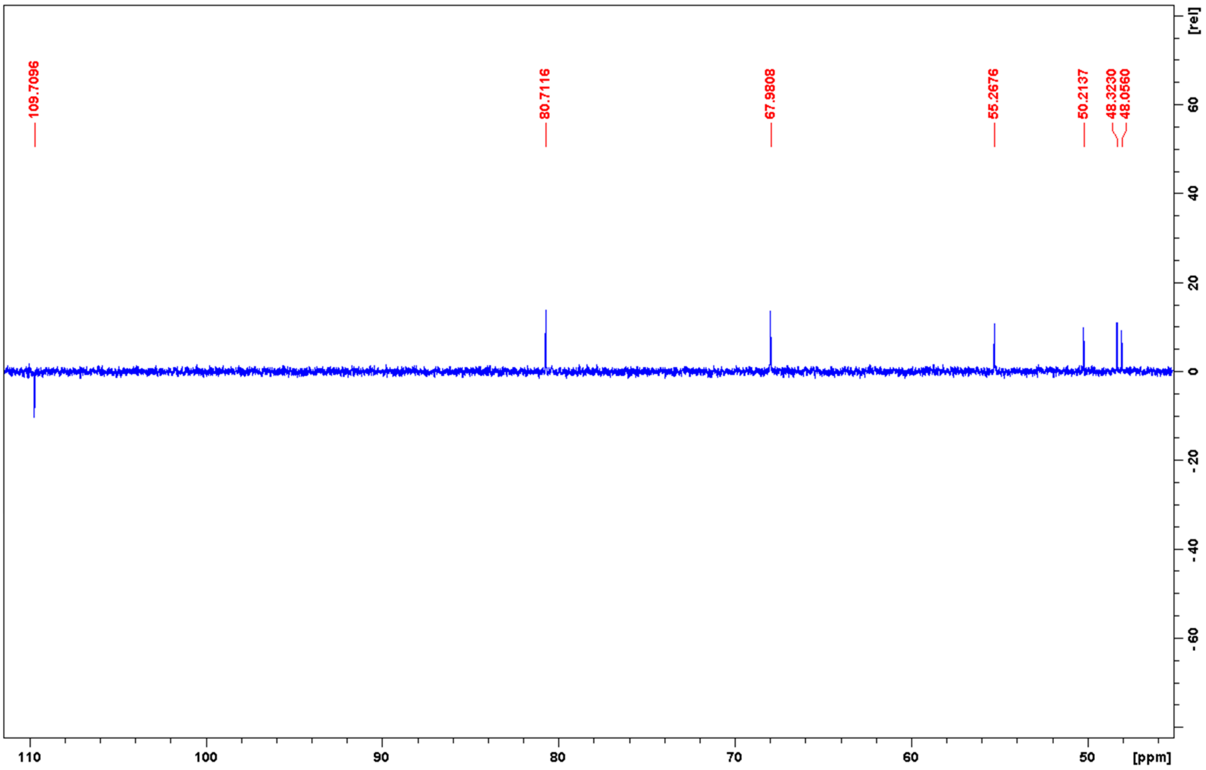

Figure S41. DEPT135 spectrum of **2** (Exp.).

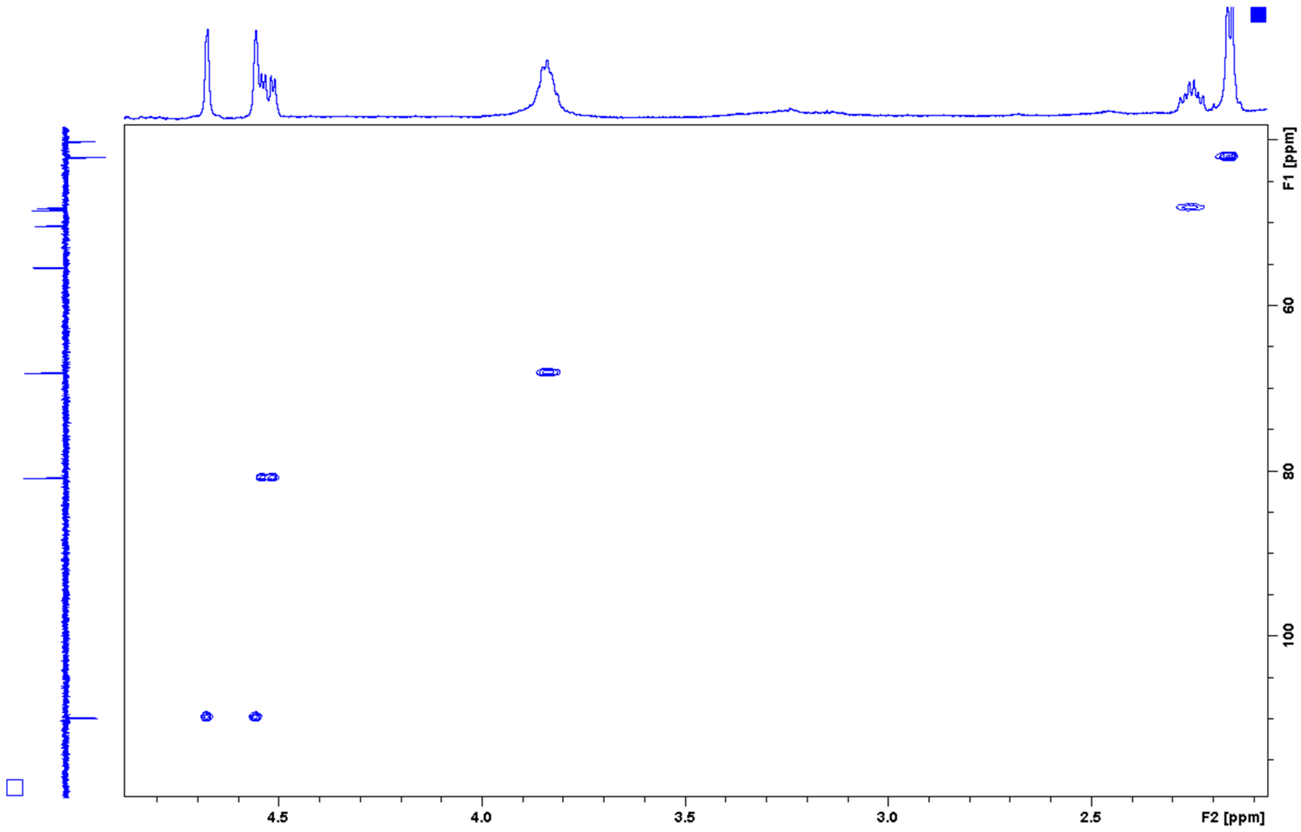

Figure S42. HSQC spectrum of **2** (Exp.).

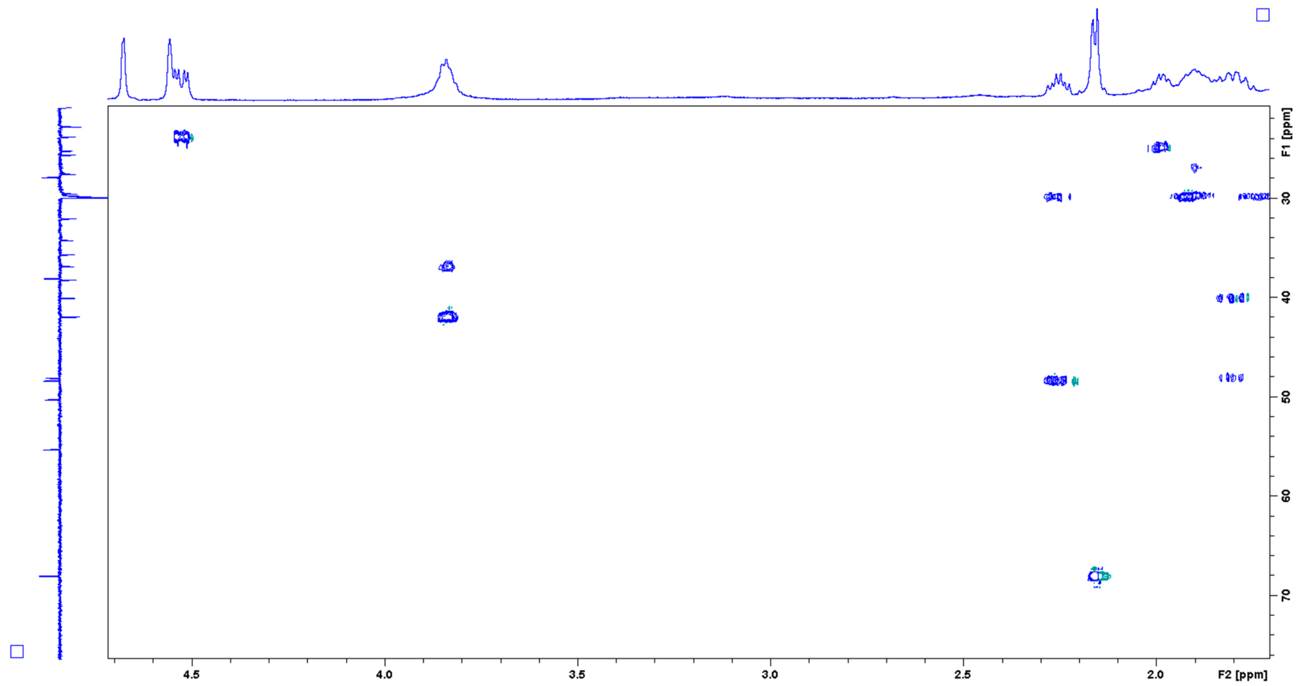

Figure S43. H2BC spectrum of **2** (Exp.).

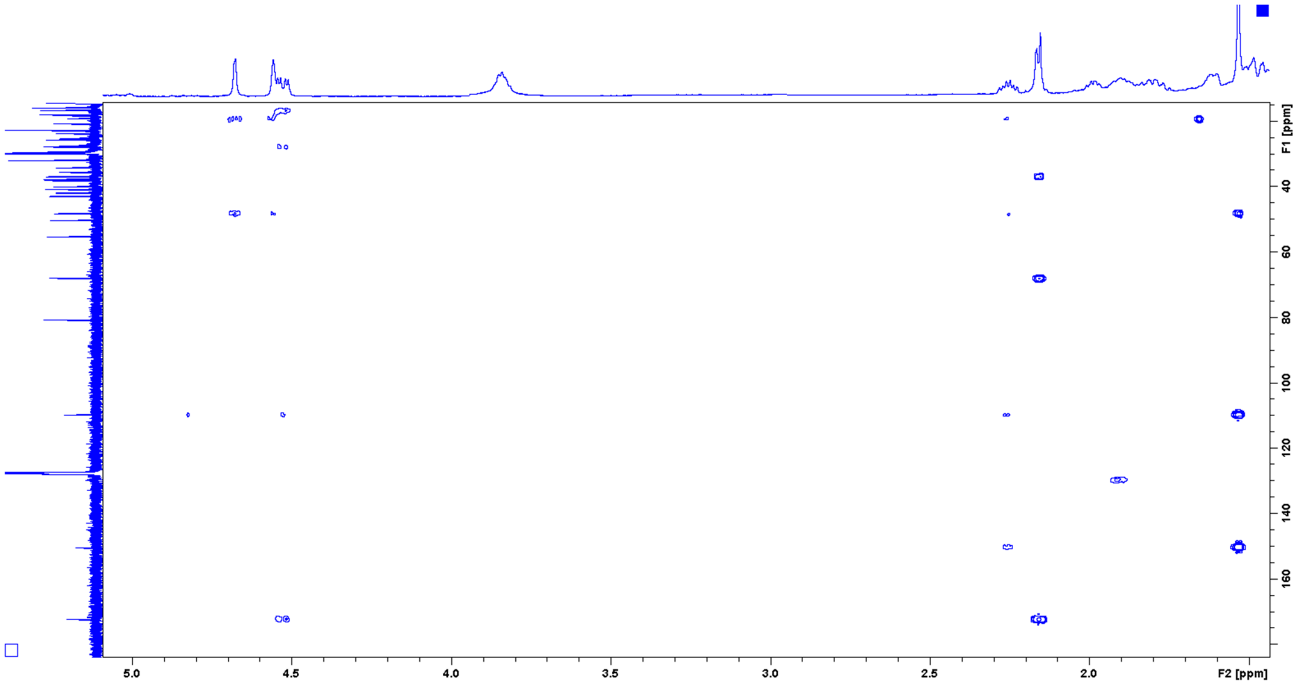

Figure S44. HMBC spectrum of **2** (Exp.).

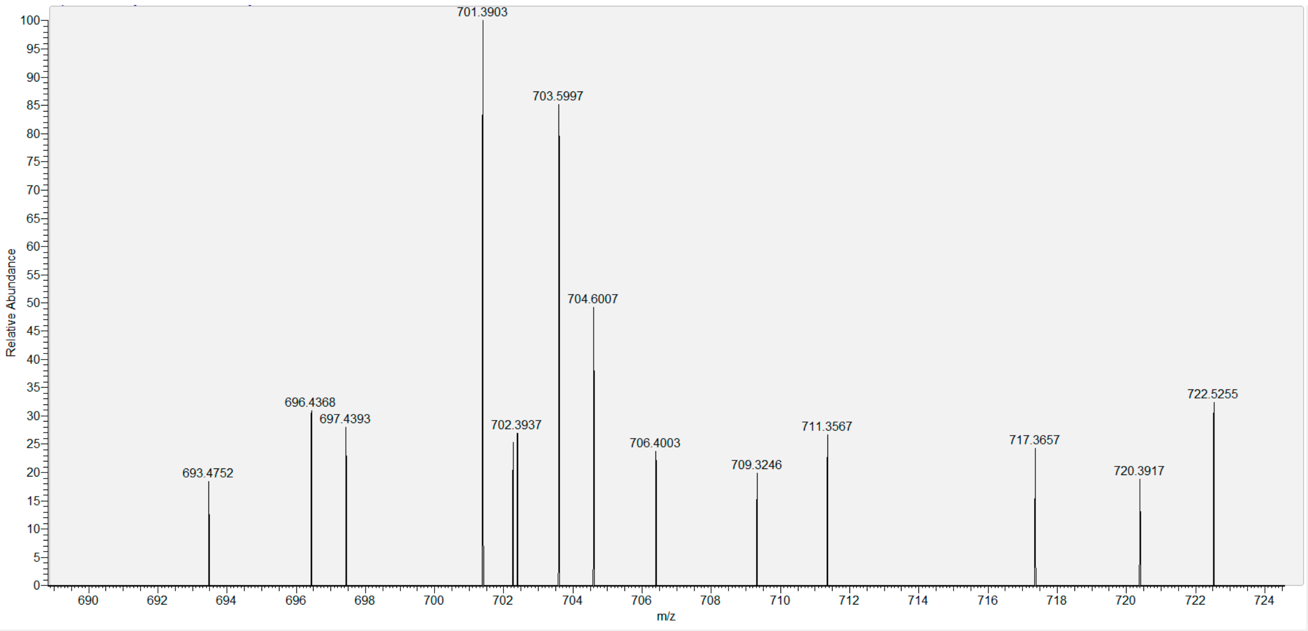

Figure S45. HRESIMS spectrum of **2** (Positive mode).

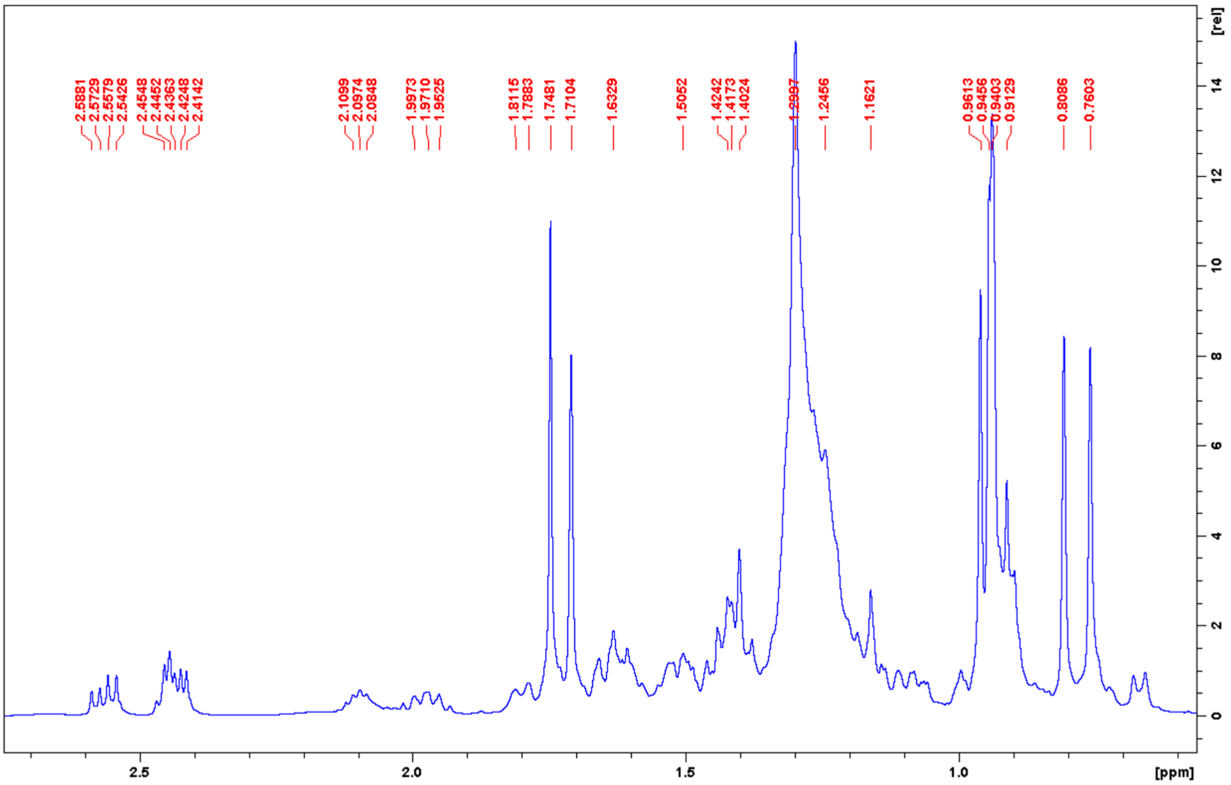

Figure S46. ¹HNMR spectrum of **2a** (Exp.).

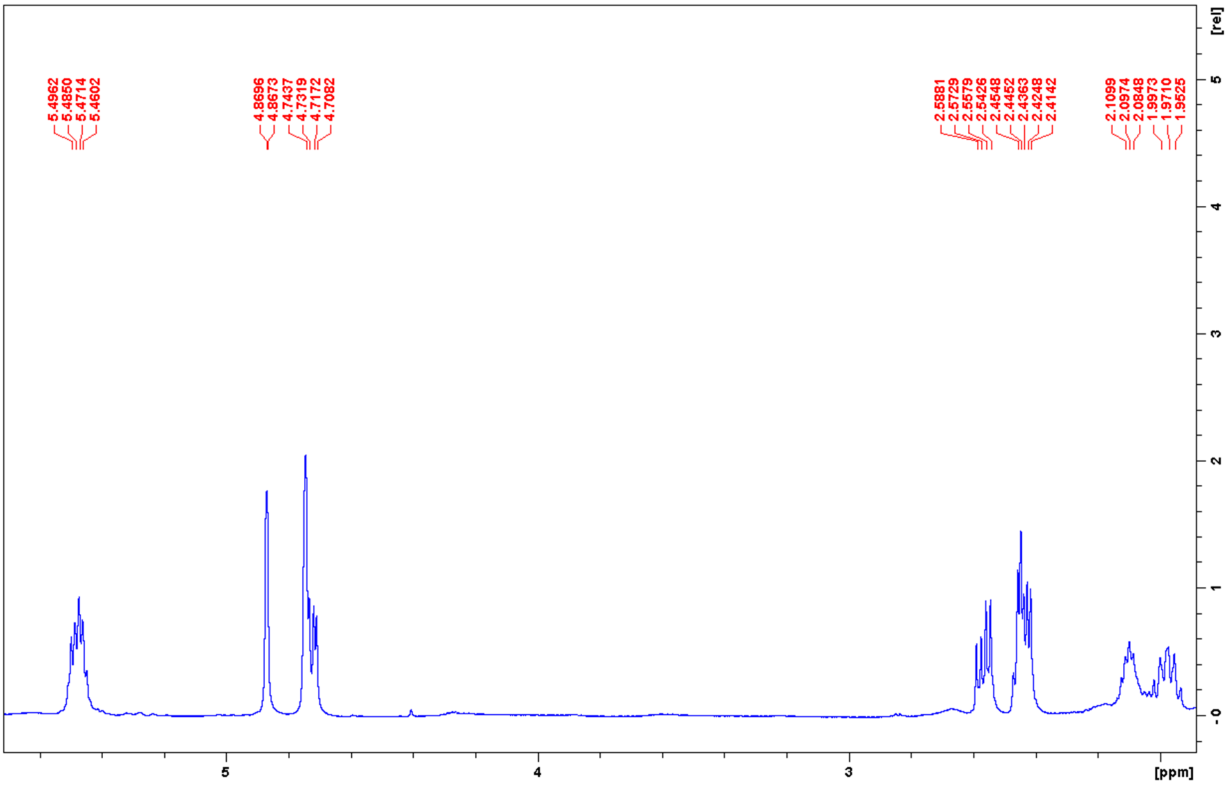

Figure S47. ¹HNMR spectrum of **2a** (Exp.).

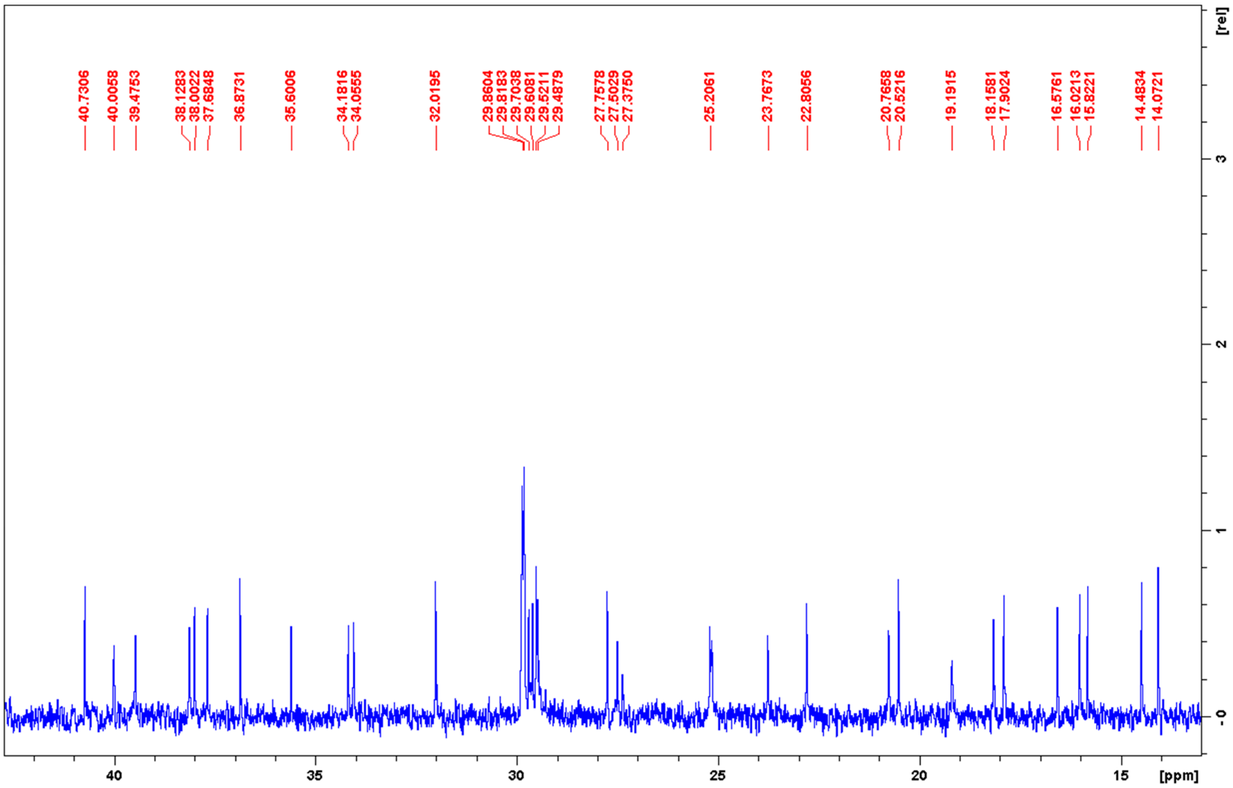

Figure S48. ^{13}CNMR spectrum of **2a** (Exp.).

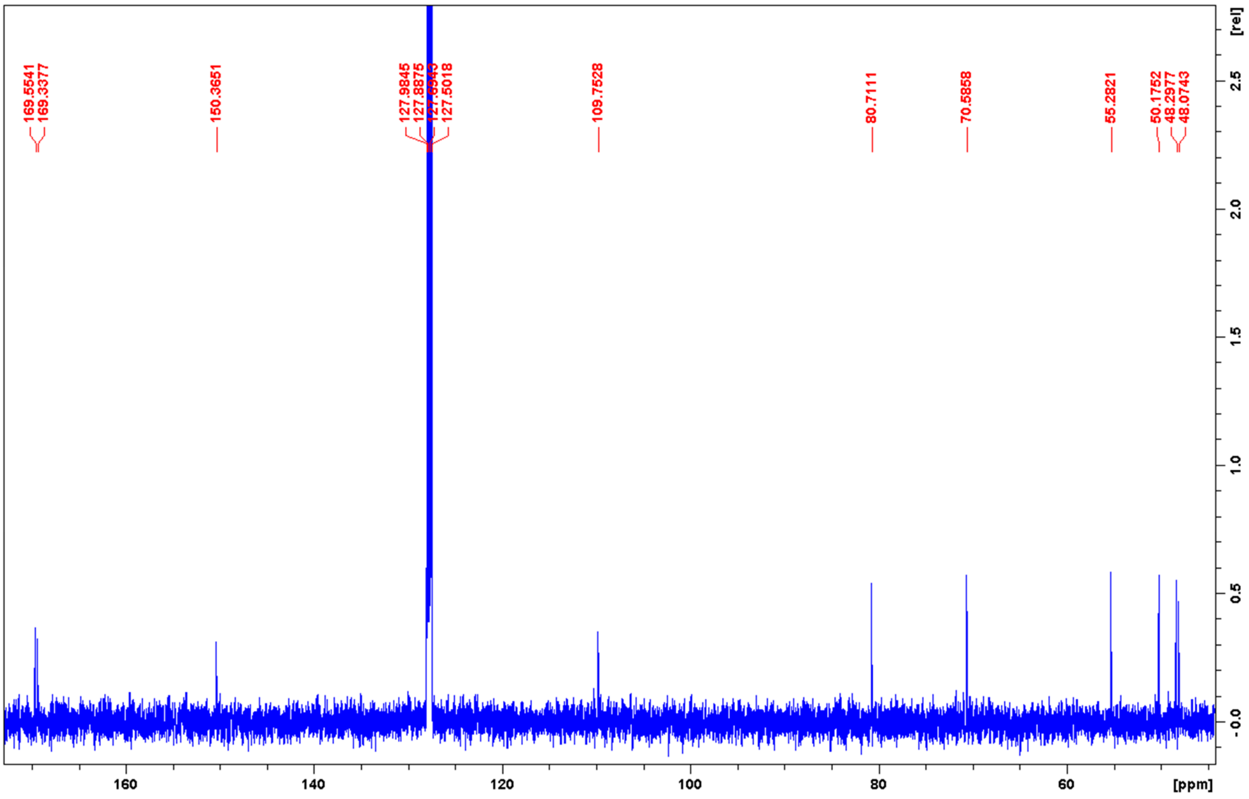

Figure S49. ^{13}CNMR spectrum of **2a** (Exp.).

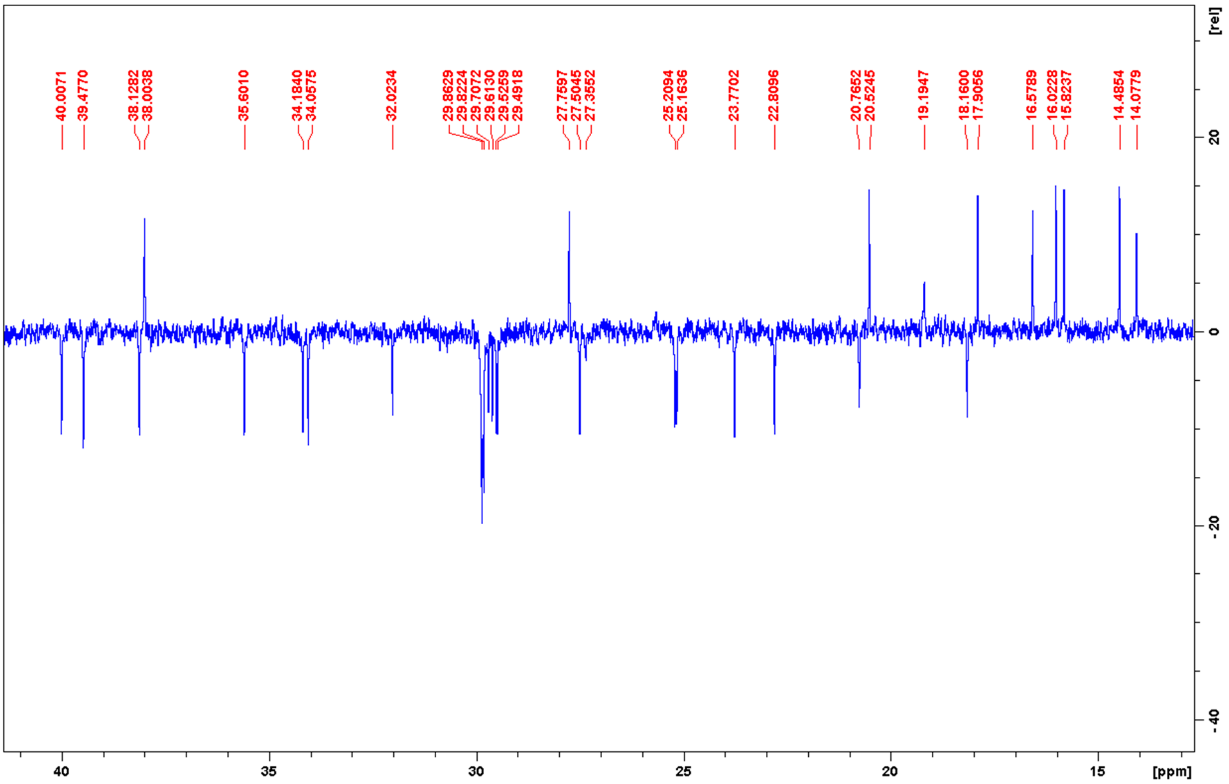

Figure S50. DEPT135 spectrum of **2a** (Exp.).

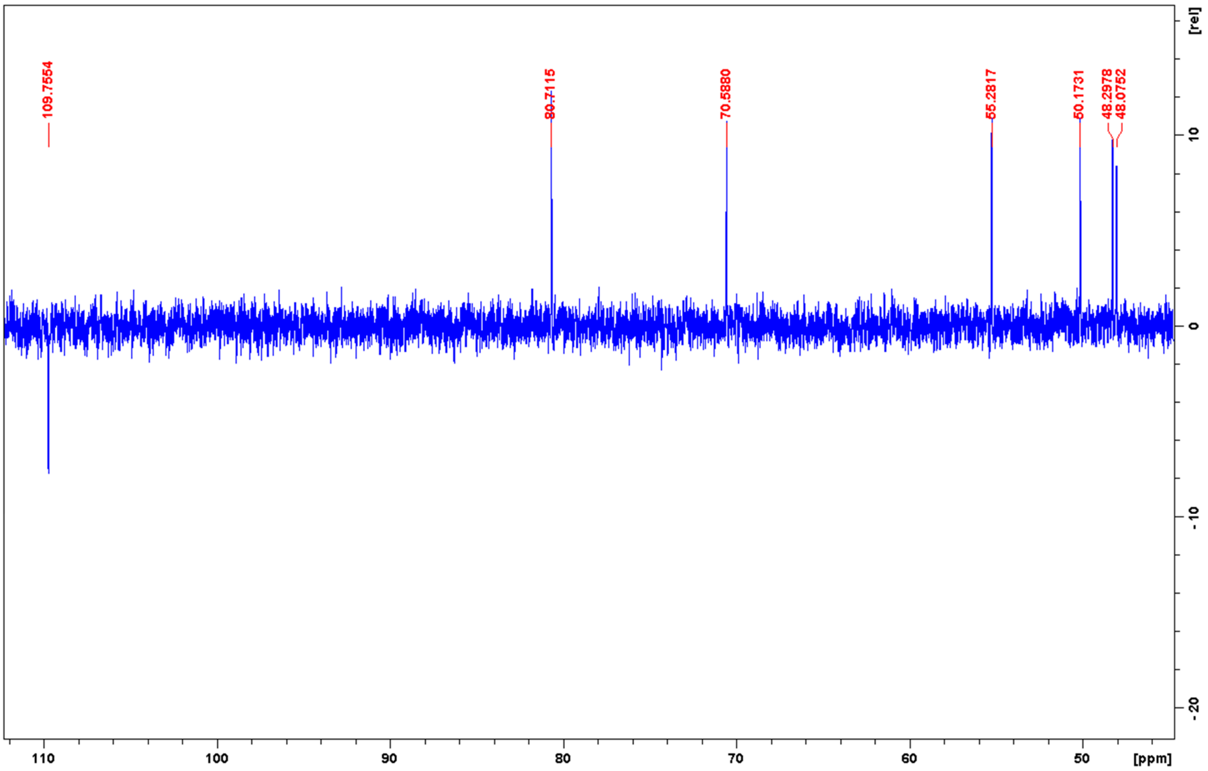

Figure S51. DEPT135 spectrum of **2a** (Exp.).

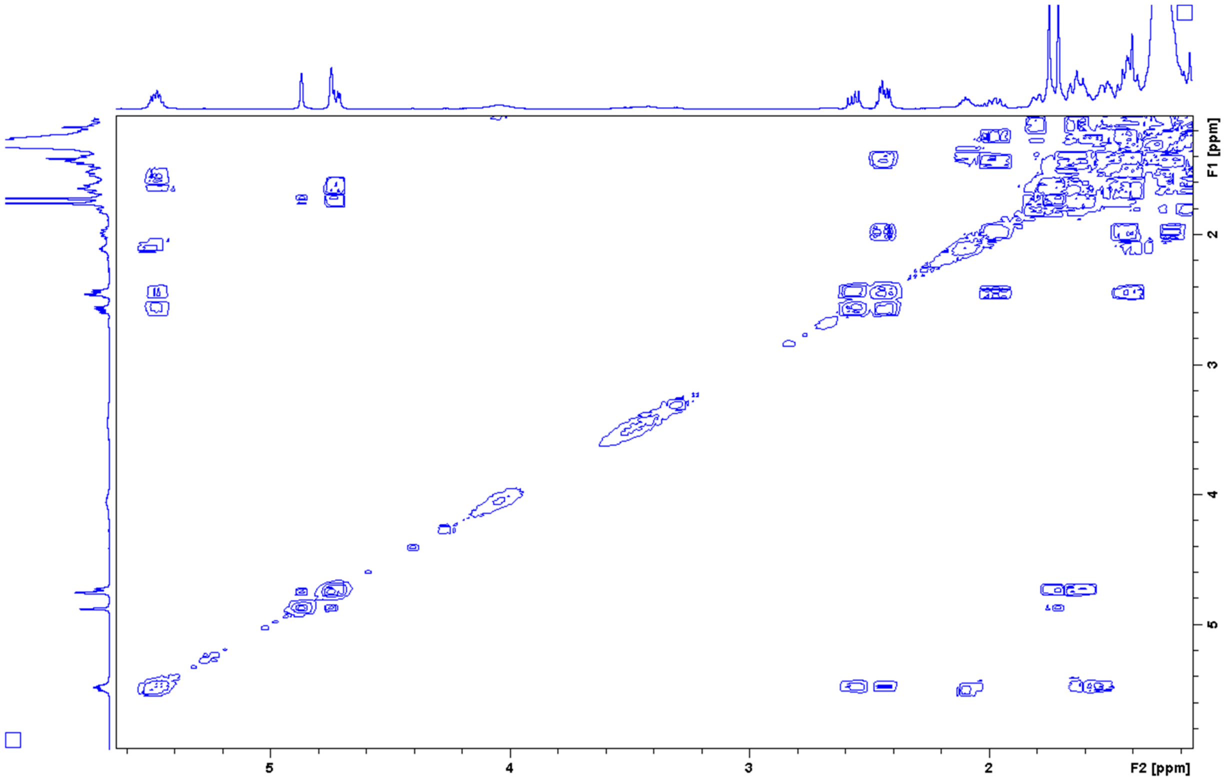

Figure S52. COSY spectrum of **2a** (Exp.).

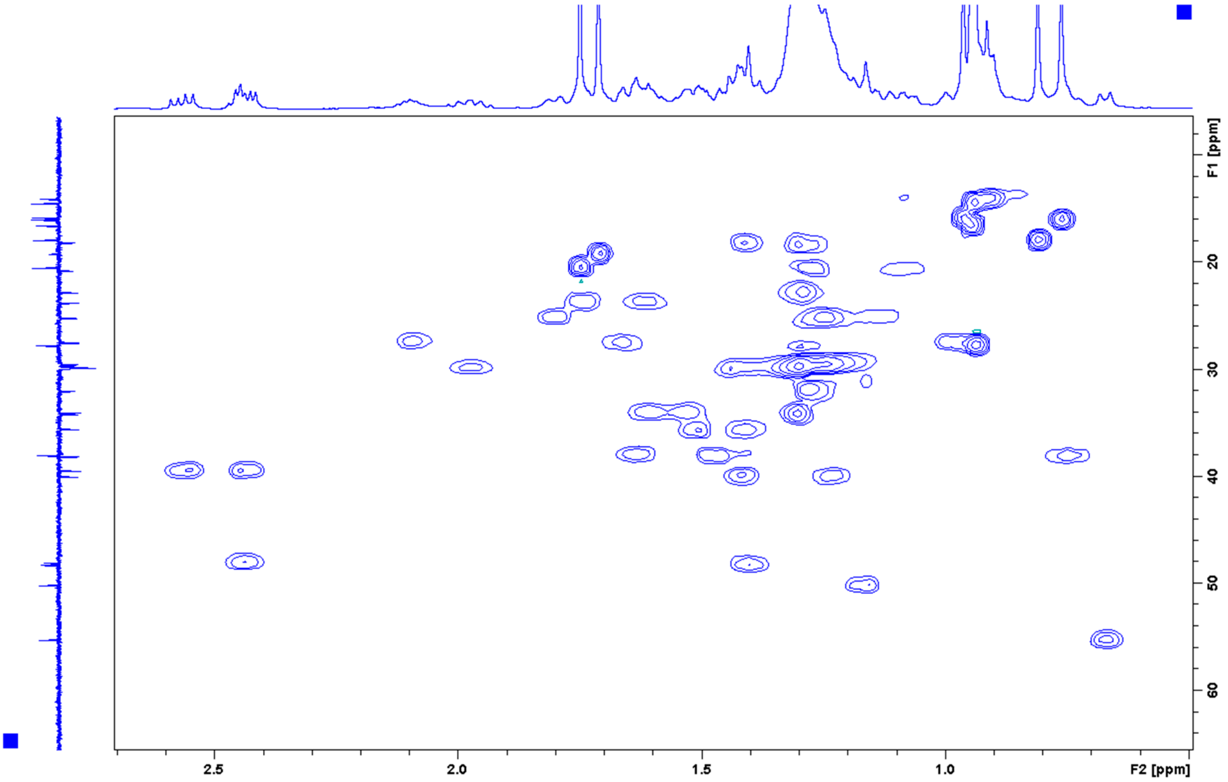

Figure S53. HSQC spectrum of **2a** (Exp.).

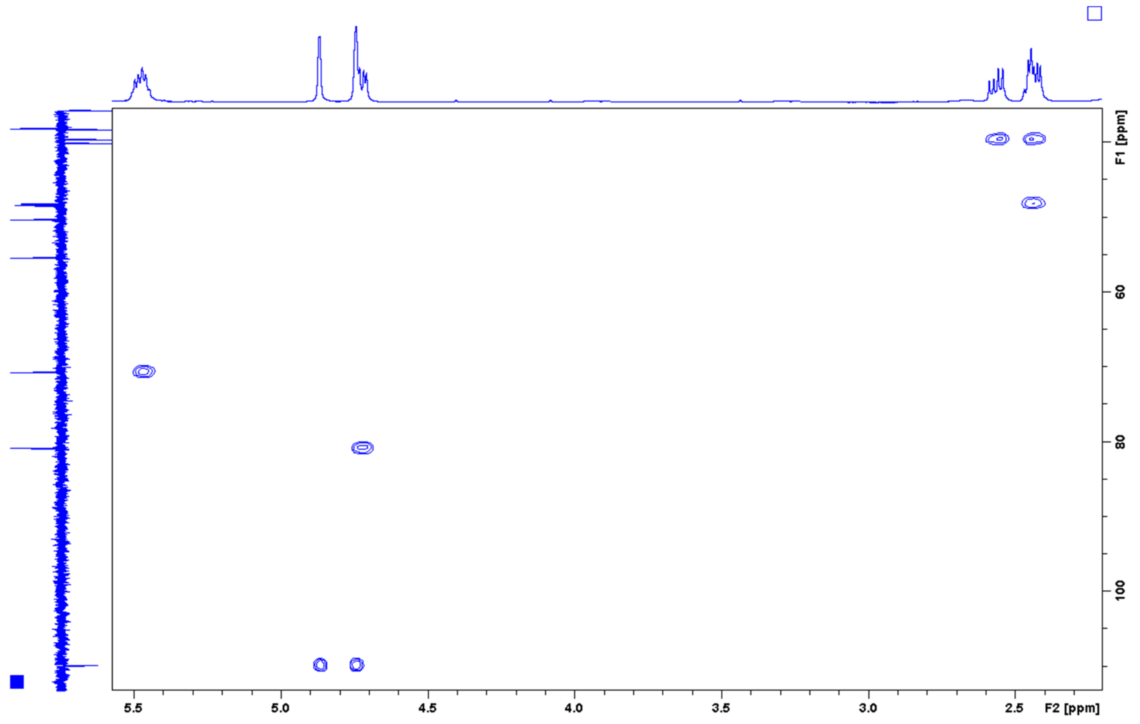

Figure S54. HSQC spectrum of **2a** (Exp.).

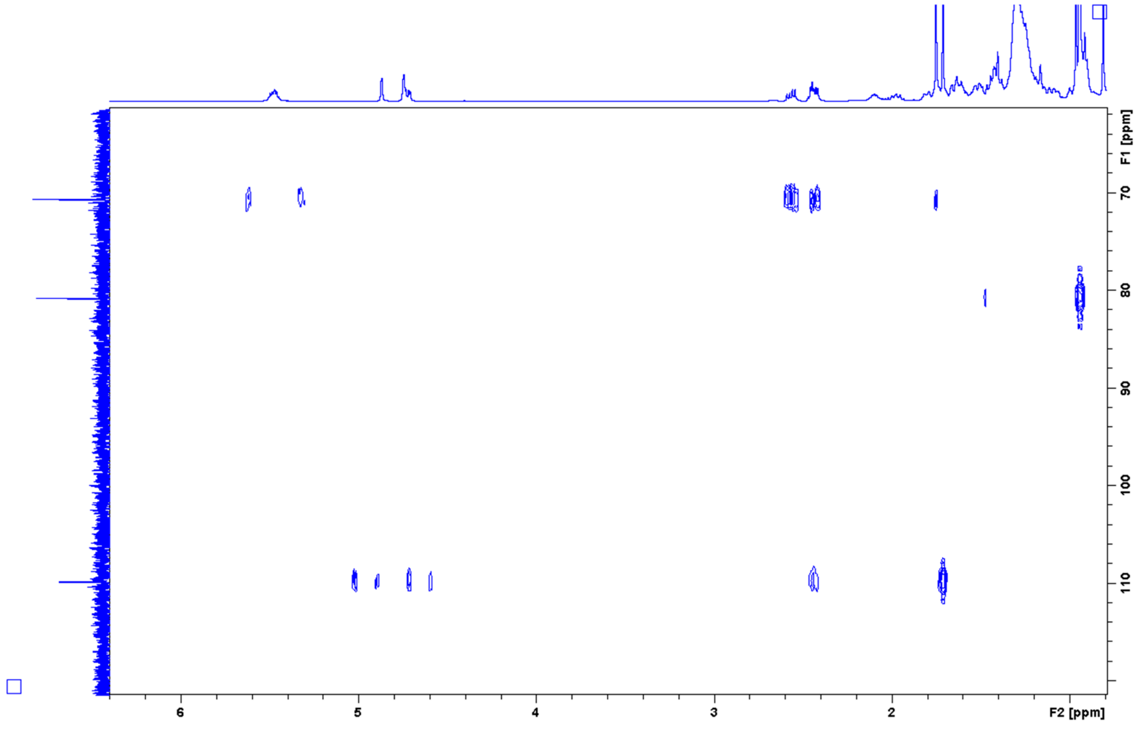

Figure S55. H2BC spectrum of **2a** (Exp.).

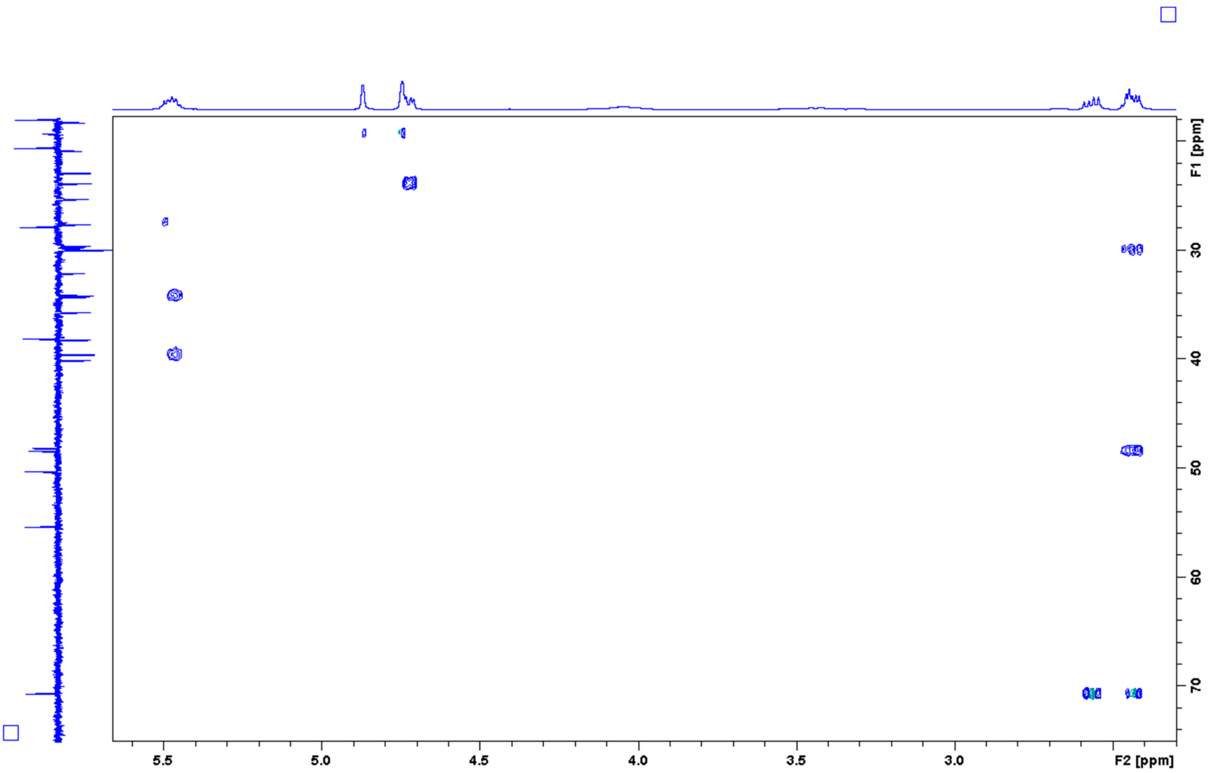

Figure S56. H2BC spectrum of **2a** (Exp.).

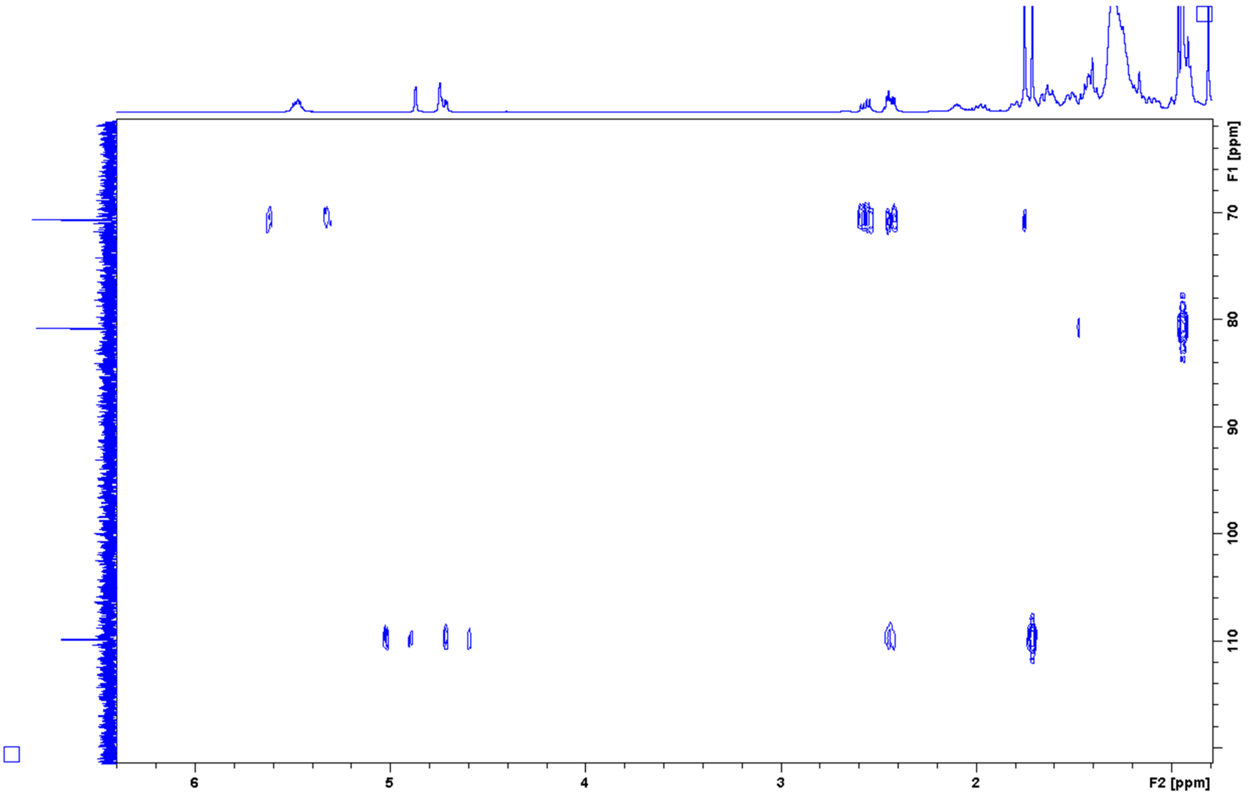

Figure S57. HMBC spectrum of **2a** (Exp.).

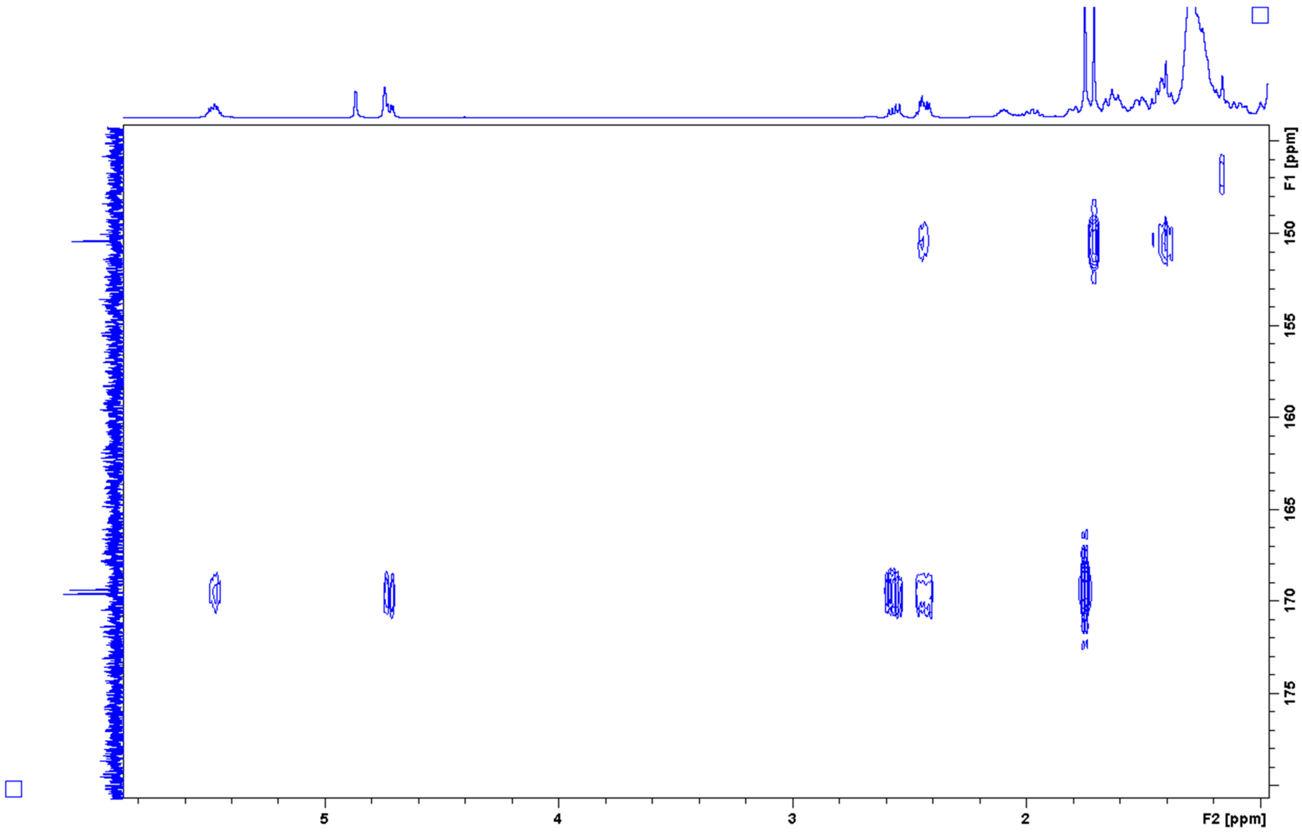

Figure S58. HMBC spectrum of **2a** (Exp.).

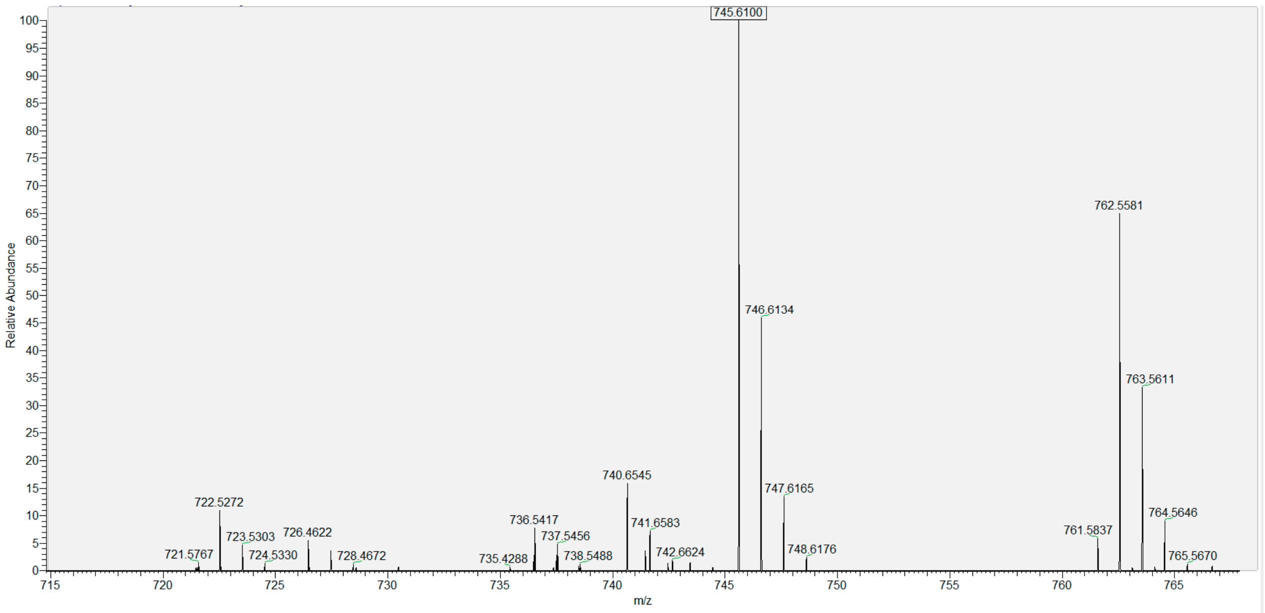

Figure S59. HRESIMS spectrum of **2**a (Positive mode).

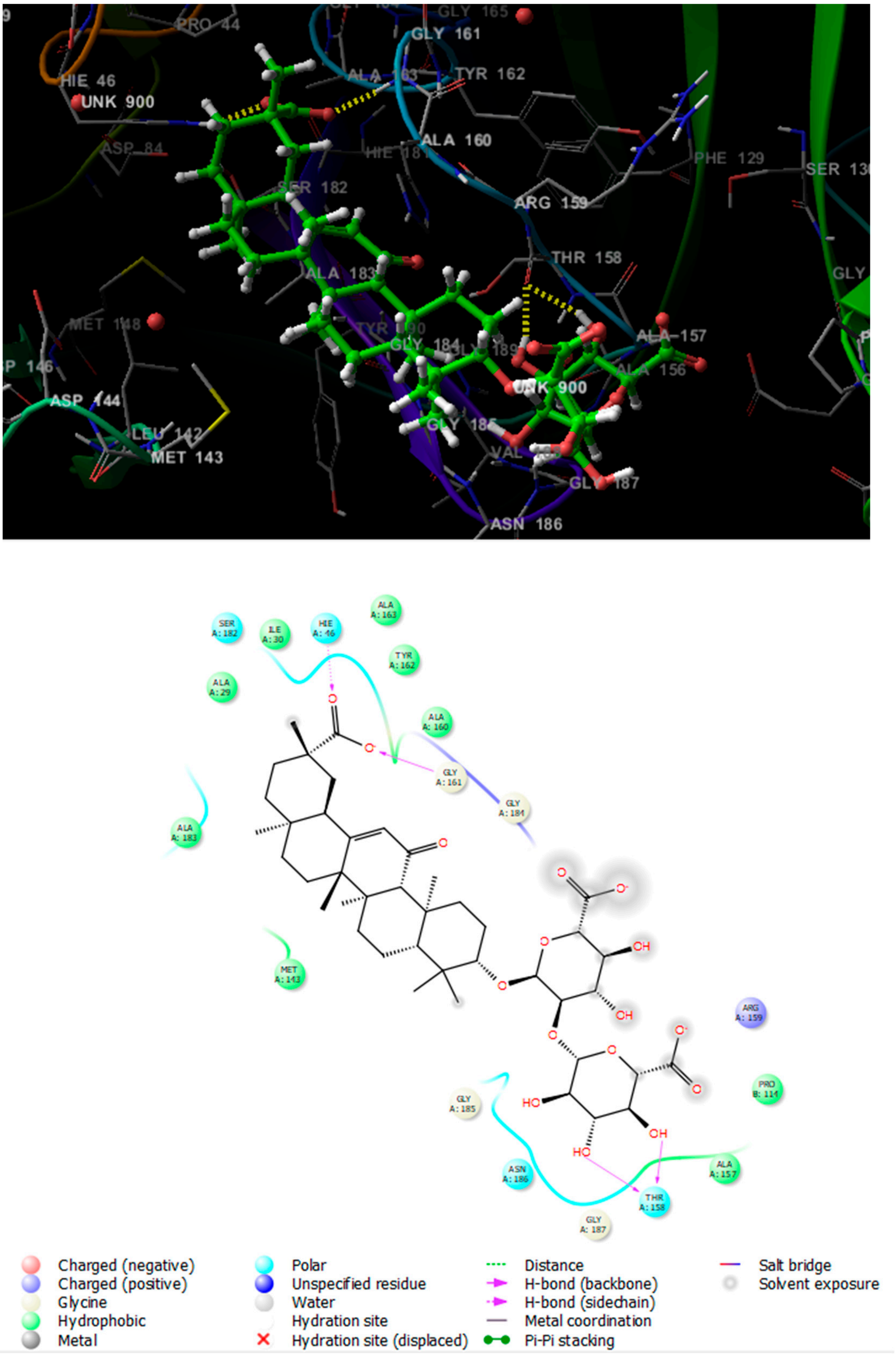

Figure S60. 3D and 2D interaction diagrams of FMDV 3C^{pro} with glycyrrhizic acid in 2D representation (lower panel).

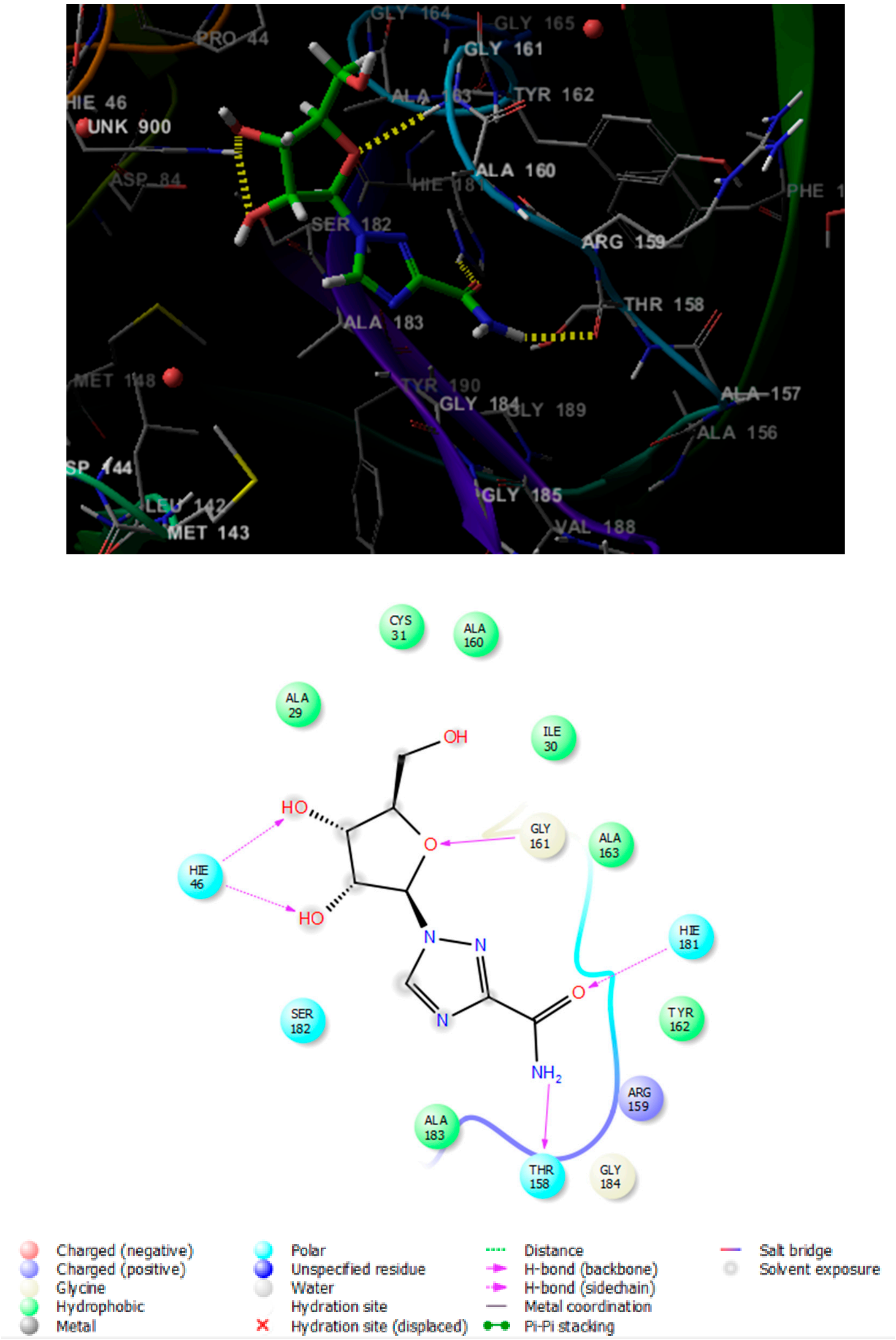

Figure S61. 3D and 2D interaction diagrams of FMDV 3Cpro with ribavirin in 2D representation (lower panel).

Table S1. Determination of total extract and fractions of *R. stricta* cytotoxicity on BHK cell.

ID	Dilution 1:2	O.D			Mean O.D	Viability %	Toxicity %	CC50
BHK	ug/ml	0.263	0.258	0.271	0.264	100	0	ug/ml
RSC	1000	0.022	0.025	0.023	0.023333	8.838384	91.16162	46.137
	500	0.025	0.023	0.024	0.024	9.090909	90.90909	
	250	0.042	0.048	0.051	0.047	17.80303	82.19697	
	125	0.06	0.072	0.077	0.069667	26.38889	73.61111	
	62.5	0.094	0.086	0.102	0.094	35.60606	64.39394	
	31.25	0.142	0.136	0.152	0.143333	54.29293	45.70707	
	15.625	0.236	0.242	0.241	0.239667	90.78283	9.217172	
	7.812	0.259	0.267	0.266	0.264	100	0	
RSE	1000	0.064	0.058	0.078	0.066667	25.25253	74.74747	699.241
	500	0.163	0.155	0.158	0.158667	60.10101	39.89899	
	250	0.268	0.252	0.269	0.263	99.62121	0.378788	
	125	0.264	0.269	0.261	0.264667	100.2525	0	
	62.5	0.257	0.269	0.253	0.259667	98.35859	1.641414	
	31.25	0.259	0.264	0.268	0.263667	99.87374	0.126263	
	15.625	0.266	0.268	0.267	0.267	101.1364	0	
	7.812	0.264	0.27	0.258	0.264	100	0	
RSH	1000	0.043	0.058	0.066	0.055667	21.08586	78.91414	593.007
	500	0.115	0.126	0.124	0.121667	46.08586	53.91414	
	250	0.195	0.204	0.213	0.204	77.27273	22.72727	
	125	0.234	0.246	0.241	0.240333	91.03535	8.964646	
	62.5	0.267	0.261	0.267	0.265	100.3788	0	
	31.25	0.27	0.262	0.259	0.263667	99.87374	0.126263	
	15.625	0.256	0.264	0.268	0.262667	99.49495	0.505051	
	7.812	0.261	0.269	0.269	0.266333	100.8838	0	
RSP	1000	0.062	0.058	0.059	0.059667	22.60101	77.39899	653.318
	500	0.136	0.141	0.138	0.138333	52.39899	47.60101	
	250	0.245	0.252	0.257	0.251333	95.20202	4.79798	
	125	0.268	0.27	0.261	0.266333	100.8838	0	
	62.5	0.261	0.272	0.264	0.265667	100.6313	0	
	31.25	0.264	0.265	0.264	0.264333	100.1263	0	
	15.625	0.268	0.263	0.2668	0.265933	100.7323	0	
	7.812	0.261	0.27	0.265	0.265333	100.5051	0	
RST	1000	0.034	0.044	0.036	0.038	14.39394	85.60606	342.362
	500	0.056	0.078	0.081	0.071667	27.14646	72.85354	
	250	0.142	0.138	0.146	0.142	53.78788	46.21212	
	125	0.246	0.252	0.247	0.248333	94.06566	5.934343	

	62.5	0.267	0.262	0.269	0.266	100.7576	0	
	31.25	0.27	0.264	0.264	0.266	100.7576	0	
	15.625	0.263	0.268	0.265	0.265333	100.5051	0	
	7.812	0.266	0.26	0.268	0.264667	100.2525	0	

Table S2. Determination of compounds **1-9** cytotoxicity on BHK cell.

ID	ug/ml	O.D			Mean O.D	Viability %	Toxicity %	SEM±	CC_{50}	MNTC
BHK	---	0.351	0.349	0.32	0.34	100	0	0.010017	ug	
**1**	1000	0.184	0.162	0.147	0.164333	48.33333	51.66667	0.010745	984.18	500
	500	0.336	0.345	0.341	0.340667	100.1961	0	0.002603		
	250	0.338	0.343	0.328	0.336333	98.92157	1.078431	0.00441		
	125	0.346	0.34	0.34	0.342	100.5882	0	0.002		
	62.5	0.335	0.353	0.331	0.339667	99.90196	0.098039	0.006766		
	31.25	0.348	0.339	0.343	0.343333	100.9804	0	0.002603		
**2**	1000	0.076	0.089	0.092	0.085667	25.19608	74.80392	0.00491	497.42	125
	500	0.142	0.159	0.172	0.157667	46.37255	53.62745	0.008686		
	250	0.295	0.301	0.312	0.302667	89.01961	10.98039	0.004978		
	125	0.341	0.326	0.34	0.335667	98.72549	1.27451	0.004842		
	62.5	0.348	0.347	0.33	0.341667	100.4902	0	0.00584		
	31.25	0.339	0.352	0.337	0.342667	100.7843	0	0.004702		
**3**	1000	0.074	0.069	0.052	0.065	19.11765	80.88235	0.006658	462.17	125
	500	0.142	0.139	0.157	0.146	42.94118	57.05882	0.005568		
	250	0.286	0.302	0.294	0.294	86.47059	13.52941	0.004619		
	125	0.328	0.351	0.343	0.340667	100.1961	0	0.006741		
	62.5	0.346	0.337	0.345	0.342667	100.7843	0	0.002848		
	31.25	0.335	0.34	0.348	0.341	100.2941	0	0.003786		
4	1000	0.214	0.196	0.225	0.211667	62.2549	37.7451	0.008452	1160.71	500
	500	0.342	0.338	0.343	0.341	100.2941	0	0.001528		
	250	0.346	0.332	0.345	0.341	100.2941	0	0.004509		
	125	0.332	0.339	0.356	0.342333	100.6863	0	0.007126		
	62.5	0.352	0.33	0.328	0.336667	99.01961	0.980392	0.007688		

	31.25	0.347	0.347	0.34	0.344667	101.3725	0	0.002333		
**5**	1000	0.084	0.096	0.101	0.093667	27.54902	72.45098	0.005044	708.18	250
	500	0.186	0.214	0.207	0.202333	59.5098	40.4902	0.008413		
	250	0.323	0.316	0.334	0.324333	95.39216	4.607843	0.005239		
	125	0.336	0.341	0.349	0.342	100.5882	0	0.003786		
	62.5	0.343	0.338	0.339	0.34	100	0	0.001528		
	31.25	0.343	0.338	0.342	0.341	100.2941	0	0.001528		
**6**	1000	0.108	0.117	0.132	0.119	35	65	0.007	818.09	250
	500	0.257	0.263	0.248	0.256	75.29412	24.70588	0.004359		
	250	0.346	0.346	0.341	0.344333	101.2745	0	0.001667		
	125	0.335	0.348	0.346	0.343	100.8824	0	0.004041		
	62.5	0.348	0.342	0.345	0.345	101.4706	0	0.001732		
	31.25	0.35	0.349	0.321	0.34	100	0	0.009504		
**7**	1000	0.095	0.088	0.105	0.096	28.23529	71.76471	0.004933	762.93	250
	500	0.248	0.263	0.258	0.256333	75.39216	24.60784	0.00441		
	250	0.324	0.319	0.338	0.327	96.17647	3.823529	0.005686		
	125	0.35	0.332	0.327	0.336333	98.92157	1.078431	0.006984		
	62.5	0.326	0.33	0.343	0.333	97.94118	2.058824	0.005132		
	31.25	0.344	0.329	0.34	0.337667	99.31373	0.686275	0.004485		
**8**	1000	0.092	0.114	0.089	0.098333	28.92157	71.07843	0.007881	174.23	125
	500	0.168	0.18	0.178	0.175333	51.56863	48.43137	0.003712		
	250	0.294	0.32	0.316	0.31	91.17647	8.823529	0.008083		
	125	0.326	0.347	0.349	0.340667	100.1961	0	0.007356		
	62.5	0.352	0.33	0.328	0.336667	99.01961	0.980392	0.007688		
	31.25	0.342	0.349	0.331	0.340667	100.1961	0	0.005239		
**9**	1000	0.096	0.114	0.136	0.115333	33.92157	66.07843	0.011566	747.39	125
	500	0.194	0.205	0.214	0.204333	60.09804	39.90196	0.005783		
	250	0.31	0.305	0.324	0.313	92.05882	7.941176	0.005686		
	125	0.328	0.339	0.349	0.338667	99.60784	0.392157	0.006064		
	62.5	0.347	0.34	0.346	0.344333	101.2745	0	0.002186		
	31.25	0.329	0.351	0.342	0.340667	100.1961	0	0.006386		
